# An open prospective study on the efficacy of Navina Smart, an electronic system for transanal irrigation, in neurogenic bowel dysfunction

**DOI:** 10.1371/journal.pone.0245453

**Published:** 2021-01-29

**Authors:** Anton Emmanuel, Ines Kurze, Klaus Krogh, Maria Elena Ferreiro Velasco, Peter Christensen, Giuilio Del Popolo, Gabriele Bazzocchi, Claes Hultling, Brigitte Perrouin Verbe, Ralf Bothig, Thomas Glott, Miguel Angel Gonzalez Viejo

**Affiliations:** 1 GI Physiology Unit, UCH London, London, United Kingdom; 2 Querschnittgelähmten-Zentrum, Klinik für Paraplegiologie und Neuro-Urologie, Zentralklinik Bad Berka, Bad Berka, Germany; 3 Department of Hepatology and Gastroenterology, Aarhus University Hospital, Aarhus, Denmark; 4 Unidad de Lesionados Medulares, Complexo Hospitalario Universitario de A Coruña, A Coruña, Spain; 5 Pelvic Floor Unit, Department of Surgery, Aarhus University Hospital, Aarhus, Denmark; 6 Neuro-Urology and Spinal Unit Dpt., Careggi University Hospital, Firenze, Italy; 7 Neurogastroenterology and G.I. Rehabilitation Unit, Montecatone Rehabilitation Institute, Imola, Bologna, Italy; 8 Karolinska University Hospital, Stockholm, Sweden; 9 Hôpital St Jacques, Nantes, France; 10 Dept. Neuro-Urology, BG Klinikum, Hamburg, Germany; 11 Sunnaas Sykehus HF, Alværn, Norway; 12 Vall d'Hebron, Unidad de Lesionados Medulares, Barcelona, Spain; University of Illinois at Urbana-Champaign, UNITED STATES

## Abstract

**Background:**

Transanal irrigation (TAI) has emerged as a key option when more conservative bowel management does not help spinal cord injured (SCI) individuals with neurogenic bowel dysfunction (NBD).

**Aim:**

To investigate the short-term efficacy and safety of an electronic TAI system (Navina Smart) in subjects with NBD.

**Design:**

We present an open, prospective efficacy study on Navina Smart, in individuals with NBD secondary to SCI, studied at three months.

**Population:**

Eighty-nine consecutive consenting established SCI individuals (61 male; mean age 48, range 18–77) naïve to TAI treatment were recruited from ten centres in seven countries. Subjects had confirmed NBD of at least moderate severity (NBD score ≥10).

**Methods:**

Subjects were taught how to use the device at baseline assisted by the Navina Smart app, and treatment was tailored during phone calls until optimal TAI regime was achieved. The NBD score was measured at baseline and at three months follow up (mean 98 days). Safety analysis was performed on the complete population while per protocol (PP) analysis was performed on 52 subjects.

**Results:**

PP analysis showed a significant decrease in mean NBD score (17.8 to 10, p<0.00001). In subjects with severe symptoms (defined as NBD score ≥14), mean NBD scores decreased (19.4 to 10.9, p<0.0001). The number of subjects with severe symptoms decreased from 41 (79%) subjects at baseline to 16 (31%) at three months follow-up. Device failure accounted for the commonest cause for loss of data. Side effects possibly related to the device developed in 11 subjects (12%). Discontinuation due to failure of therapy to relieve symptoms was reported by 5 subjects (6%).

**Conclusion:**

Navina Smart is effective for individuals with NBD, even those with severe symptoms; long-term data will follow. Whilst there were some device problems (addressed by the later stages of subject recruitment) the treatment was generally safe.

**Clinical trial:**

(ClinicalTrials.gov number NCT02979808)

## Introduction

Spinal cord injury (SCI) is a life-changing event with a range of physical and psycho-social sequelae, which are chronic and require long-term management and monitoring. Spinal cord injury, both traumatic and non-traumatic, has an estimated prevalence of over 2.5 million worldwide [1]. Symptoms of constipation and faecal incontinence are common after SCI, and are termed neurogenic bowel dysfunction (NBD). Up to 95% report constipation [2] and 75% have experienced episodes of faecal incontinence [3]. These symptoms also seem to deteriorate with time [4], an important factor given the advances in survival after SCI. In terms of adverse effects on quality of life, bowel dysfunction is rated very highly by individuals with SCI, ranking second only to their loss of mobility in some studies [2, 5], while bowel incontinence is reported as the greatest source of social discomfort in others [6, 7]. Management of NBD is often challenging [8], with pharmacological approaches to treat one aspect causing exacerbations of the other (e.g. use of laxatives to treat constipation resulting in incontinence; and anti-diarrhoeals readily exacerbating constipation).

Transanal irrigation (TAI) has emerged as an effective [8, 9] and cost-favouring approach to management of NBD [10]. The mechanism of TAI is to assist the evacuation of faeces from the bowel by introducing water into the colon and rectum through the anus in order to induce a reflex colorectal voiding. The water is introduced using a single-use catheter or cone, and after the device is removed, the contents of the rectum and some of the more proximal colon is voided [11]. Most devices require manual compression of a pump to introduce water from the water reservoir into the bowel. This does not suit all individuals with SCI, particularly those with limited manual dexterity or impaired coordination. Navina Smart (Wellspect Healthcare) is the first electronic system for transanal irrigation (TAI) and consists of an electronic control unit, pump and smartphone app. The control unit permits more accurate monitoring of irrigation parameters. The connection to the Navina Smart app makes monitoring of treatment more predictable and accurate, and as such may improve treatment adherence. We have previously reported on the safety and efficacy of Navina Smart in individuals refractory to TAI therapy with other systems [12]. We now report an open, prospective, multi-centre study of Navina Smart, with the primary aim being to evaluate change in NBD symptoms after three months.

## Material and methods

### Study design

We undertook an open, prospective, non-controlled, multicentre study in a SCI population of 89 subjects with confirmed NBD from ten centres in seven European countries. The plan was to enroll 150 subjects, patients from the clinics register, to achieve 80 evaluable subjects at 3 months analysis with 90% power (Student’s t-test, 2-sided) and detection of minimal important difference in NBD of 0.8, S1 Protocol. Subjects were expected to be in the study for a total of 12 months, with three scheduled site visits (Baseline, 3 months and 12 months follow up) and ad hoc telephone follow-ups performed after baseline visit until the subject had an optimal routine. The presented analysis includes the baseline and 3 months visits from the study NAV-0001 with ClinicalTrials.gov number NCT02979808.

### Subject enrollment criteria

Before screening, all individuals gave written informed consent for their participation in the study at a visit to the clinic, in accordance with Good Clinical Practice standards and the Declaration of Helsinki. Eligible males and females subjects were above 18 years old, with confirmed chronic spinal cord injury (SCI), either traumatic or non-traumatic, of at least 3 months duration. All subjects had to be naïve to TAI treatment and with a confirmed NBD score ≥10 despite conservative therapy. Faecal impaction was excluded in all subjects at baseline by clinical examination (abdominal and digital rectal examination) by the clinician ahead of recruitment to the trial. Subjects were required to not have any confirmed or suspected diagnosis of anal or rectal stenosis, history of pelvic radiotherapy, colorectal surgery within the preceding 3 months, active inflammatory bowel disease, acute diverticulitis, severe diverticulosis, colorectal cancer, ischemic colitis, history of life-threatening autonomic dysreflexia, bleeding disorders, unspecified peri-anal conditions. Subjects had to be able to manage a smartphone or tablet.

### Study device

The Navina Smart system (CE-marked) is intended for TAI and consists of a hydrophilic rectal catheter with an in-/deflatable balloon for retention, tubing, connectors, a water container, a handheld electrical control unit (ECU) and a smartphone/tablet app. The control unit regulates the pressure to the water container, the water volume as well as inflation and deflation of the balloon on the rectal catheter. The ECU registers and stores data (balloon size, water volume, water flow rate as well as start and stop time of irrigation) during each irrigation that can, by Bluetooth, be sent to the Navina Smart app and then forwarded to the healthcare provider.

At the first visit the responsible investigator/healthcare provider trained the subject in using the Navina Smart system including the app for continuous use at home. The process for initiation of the therapy with regard to frequency and timing of use was individually assessed based on each subject’s clinical need as mutually agreed with the investigators. Adherence to therapy was assessed by study nurses during phone calls until stable therapy (namely until the settings such as balloon size, water volume and flow rate were providing self-reported consistent effective irrigation for the subject) and again at the 3 months visit. A treatment schedule providing guidance on the settings of the device was used by the investigators if needed, S1 Appendix.

### Patient reported outcome

A user friendly version of the NBD score (meaning that the wording was amended from the original to fit local language, and this was approved by the developer [13]), was used to assess efficacy of TAI with Navina Smart. The NBD score could be completed in the app and sent to the healthcare provider. In addition to the NBD score, a study-specific subject-reported questionnaire (S1 Questionnaire) was designed to record current bowel management, training received on TAI, time needed for bowel management, handling of Navina Smart including the app and satisfaction with bowel management. TAI safety data was collected continuously during the study and at the follow-up visit.

### Statistical analyses

Descriptive statistics were used to analyze and present data, that is, number of subjects (N), mean/median, variability (standard deviation (SD)/min-max) for continuous data and frequencies and percentages for categorical data. No missing data were replaced or estimated. Statistical tests were performed with non-parametric methods (the Wilcoxon signed rank test and the Binomial/McNemar test to compare paired observations) and therefore normality tests were not needed. Proportions were tested by the McNemar’s test if the number of non-equal answers between the assessment time-points were greater than 10 and by the binomial test if less than or equal to 10. The Spearman rank correlation coefficient was used for estimating the degree of linear correlation. A two-sided p‐value below 0.05 was regarded as statistical significance. All statistical tests were performed using the statistical software StatXact.

### Ethics

The study protocol was approved by the ethics committees/institutional review boards for all participating study centers (NAV-0001, Title: An open, qualitative, prospective, multicenter trial of a novel transanal irrigation system in spinal cord injured patients) as follows: Denmark Region Midtjylland number 58076 sagsnumber 1-10-72-224-16; France CPP Ouest IV ref 26/16, CNIL 1968367, CNOM 2007–454, ANSM DMTCOS/DMCARD/ML/2016-A00857-44/MS1; Germany Landesärtzekammer Thüringen 55301/2016/63 and Ärztekammer Hamburg PV5311; Italy Comitato Etico Regionale per la Sperimentazione Clinica della Regione Toscana 10444_spe and Servizio Sanitario Regionale Emilia-Romagna 112808; Norway Regionale komiteer for medisinsk og helsefaglig forskningsetikk 2016/1494/REK sör-öst C; Spain Comité Ético Vall D’Hebron PR(ATR)140/2016; UK the Yorkshire & The Humber—Bradford Leeds Research Ethics Committee REC number 16/YH/0288.

## Results

### Subject enrollment

**Fig 1:**
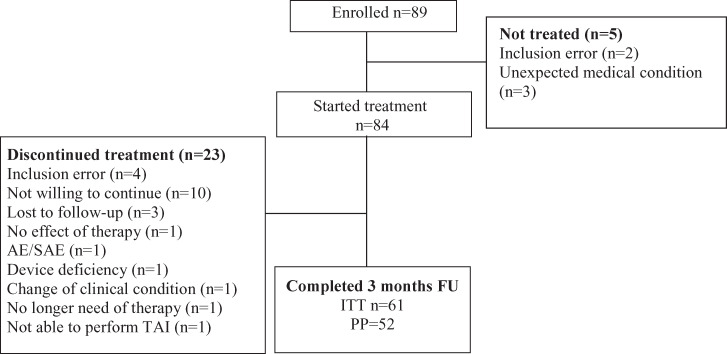
Disposition of study subjects. A total of 89 subjects were enrolled in the study while 84 started treatment and after 3 months 28 subjects were withdrawn (33% drop-out (28/84)). (n, number; AE/SAE, adverse event/serious adverse event; ITT, intention to treat; PP, per protocol, FU, follow up).

Between Oct 2016 and Sep 2018, 89 subjects were enrolled in the study (Denmark n = 4, France n = 7, Germany n = 15, Italy n = 15, Norway n = 4, Spain n = 26, United Kingdom n = 18). Recruitment stopped after 89 subjects were enrolled, and after the recruitment period had been extended by 12 months. The disposition of study subjects is shown in Fig 1: after the first 3 months 28 subjects were withdrawn or excluded from the study and 9 had major protocol deviations (major visit window deviations), which excluded them from the per protocol (PP) analysis data set. Out of the 28 withdrawn subjects, 6 were withdrawn from the study due to inclusion error whereof 2 did not start treatment (i.e. the subjects did not fulfill the criteria for study participation). 3 subjects did not start treatment since they at the first visit had a medical condition that could interfere with the therapy. Device failure (due to either mechanical failure of the pump, or failure of communication between the handset and electronic control unit) was the most frequent cause for loss of data followed by patient withdrawing consent to continue study participation. At the time of this partial clean file analysis (1^st^ of March 2019), all subjects had completed the 3 months visit and there were 21 subjects ongoing and 20 subjects had completed the full study. Accordingly, data presented are based on 52 subjects that followed the protocol (PP analysis set) and treatment safety are based on 84 subjects (5 enrolled subjects did not use the device). Concomitant medication was recorded, and two patients started treatment for neurogenic bladdder during the course of the study. No patients discontinued therapy for bladder dysfunction.

The demographic and injury-related characteristics of the study population is shown in Table 1.

**Table 1:** Demographic and injury-related characteristics. The demographic and injury-related characteristics of the 52 subjects in the per protocol data set.

Demographic characteristic, N 52			
Sex, N (%)	Male	37	(71%)
	Female	15	(29%)
Age (years)	Mean (SD)	47.6	(14.1)
	Range	18–77	
ASIA score, N 51*			
	A	23	(44%)
	B	7	(13%)
	C	8	(15%)
	D	13	(25%)
Hand function, N 51*			
No restriction		30	(58%)
Unilateral impaired		5	(10%)
Bilateral impaired		10	(19%)
No hand function		6	(11%)

*1 subject with missing data.

### NBD score and satisfaction

There was a significant reduction in NBD score seen after 3 months use of Navina Smart, with mean NBD score reduced from 17.75 ± 4.87to 9.94 ± 5.26 (p<0.0001). In 41 subjects with severe symptoms (defined as NBD score equal to or above 14), NBD scores decreased from 19.39±4.10 to 10.93±5.28 (p<0.0001).

**Fig 2:**
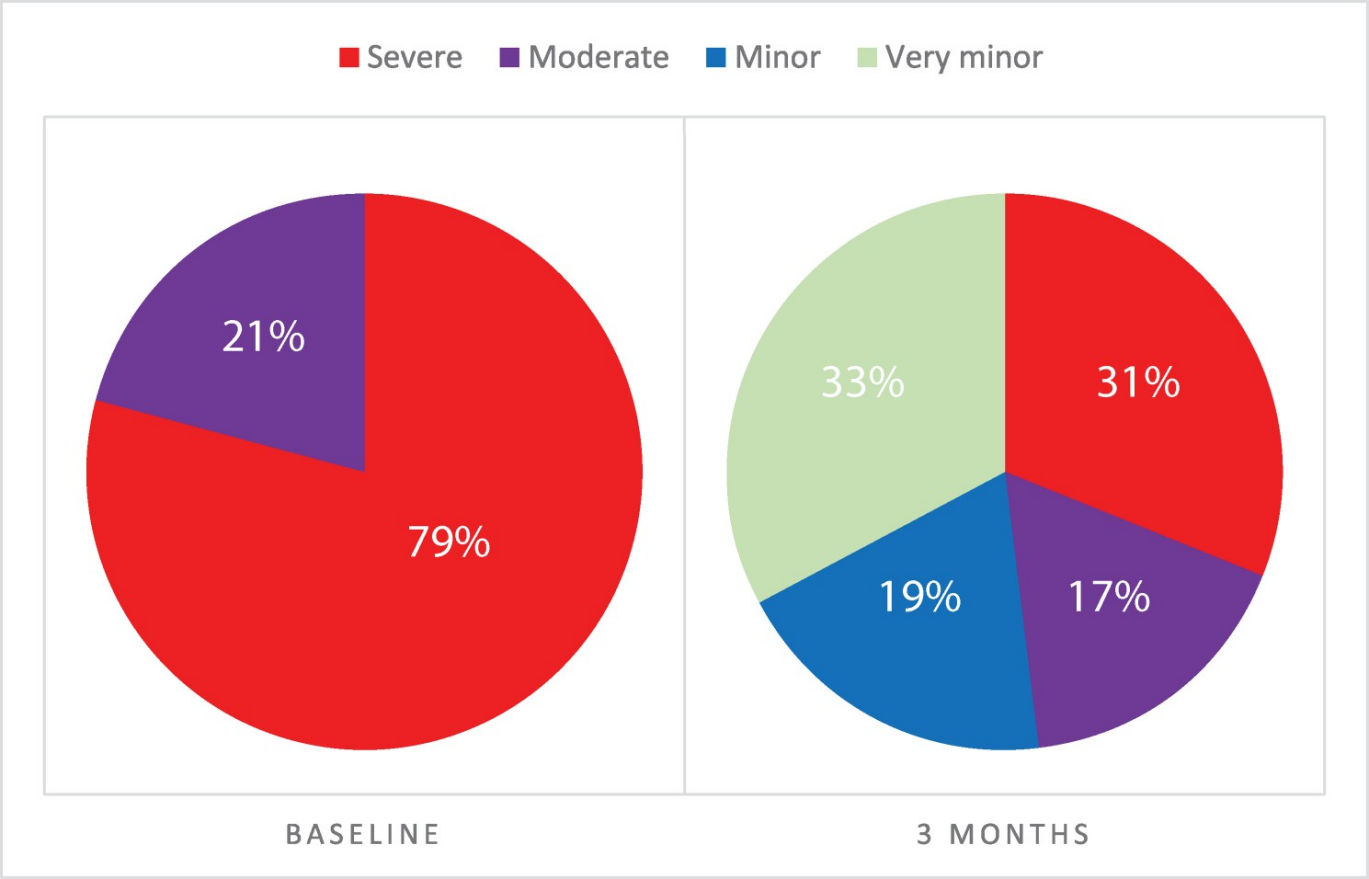
Change in NBD severity. The were 41 subjects with severe NBD symptoms at baseline visit and after 3 months use of Navina Smart the number had decreased to 16 subjects with severe NBD symptoms. A third of the subjects had very minor NBD symptoms after 3 months in the study.

The proportion of subjects with severe symptoms decreased from 79% (n = 41) at baseline to 31% (n = 16) at three months follow-up, Fig 2. In total, there were 17 subjects that after 3 months of TAI had very minor NBD symptoms (NBD score 0–6), while 10 had minor NBD symptoms (NBD score 7–9).

**Fig 3:**
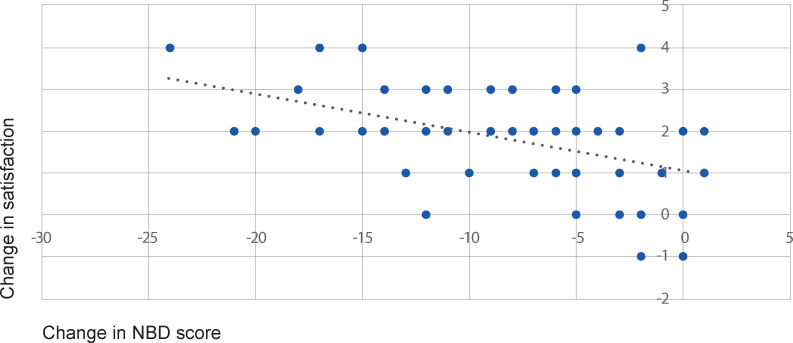
Correlation of NBD score and satisfaction with current bowel management. Change in NBD score (x axis) after 3 months use was correlated with change in satisfaction with current bowel management (y axis) after 3 months use of TAI. The Spearman rank correlation coefficient was -0.46, p = 0.0006 between decreased NBD score and increased satisfaction with subjects’ bowel management.

Subjective satisfaction with bowel management, measured on a 5 point scale (where a higher value represented greater satisfaction) increased from mean 1.85 (±0.78, range 1–4) at visit 1 to mean 3.67 (±0.98, range 1–5) after 3 months (p<0.0001). There was a correlation between NBD score and satisfaction with bowel management (p = 0.0006), Fig 3. The correlation coefficient of 0.46 is considered to indicate a moderate relationship.

Subjects in this cohort were naïve to TAI therapy, and equivalent proportions were completely or partially satisfied with Navina (81%, 42/52, 95% CI 67% - 91%) as were satisfied with TAI as a novel therapy (83%, 43/52, 95% CI 70% - 92%). Dissatisfaction levels were also similarly low with regard to Navina specifically and TAI as a therapy (3/52 [6%] vs 4/52 [8%], respectively).

### Bowel management

The subjects had significantly reduced the time spent on their daily bowel management from mean 73.5 min to 46.4 min (range visit 1 0–360 min (±61.9), visit 2 10–120 (±20.5), p = 0.0007) after 3 months use of Navina Smart. There was a decrease in the need of other methods for bowel management besides TAI from a total of 225 (all methods, all subjects) at visit 1 to 158 at visit 2, see Table 2. This equates to a reduction from mean 4.3 to 3.0 after 3 months of using TAI. In subjects with faecal incontinence the number of subjects that needed pads did not significantly decrease (17 subjects at visit 1 and 11 subjects at 3 months). Of those 17 respectively 11 subjects that used pads, the number of pads used did not decrease significantly either (mean 11.2±6 /median 10 pads per week and subject at visit 1 to mean 11.3±7 /median 7 pads per week and subject after 3 months).

**Table 2:** Bowel management. Subjects were asked which methods they use as a part of their current bowel management at baseline and after 3 months use. The subjects completed the questions themselves at both visits. Total count of number of methods used for bowel management decreased after 3 months in the study.

Which method(s) do you usually use as a part of your current bowel management?	Visit 1 (Baseline) N = 52	Visit 2 (3 months) N = 52	Difference in number of subjects (visit 2 –visit 1)
Diet and fluids	30	34	-4
Stool softeners	28	23	5
Spontaneous/voluntary bowel emptying	10	7	3
Digital rectal stimulation	32	17	15
Digital removal of faeces	35	12	23
Micro-enema laxatives	14	5	9
Rectal suppository laxatives	17	3	14
Leaning forward during bowel emptying	24	23	1
Massage of abdomen	30	33	3
Other	5	1	4
None	0	1*	-1
Total	225	158	
Mean per subject (total number of methods divided with number of subjects)	4.3 ±1.65	3.0±1.43	

*Not included in total and mean count.

### Training in therapy and Navina Smart app

**Fig 4:**
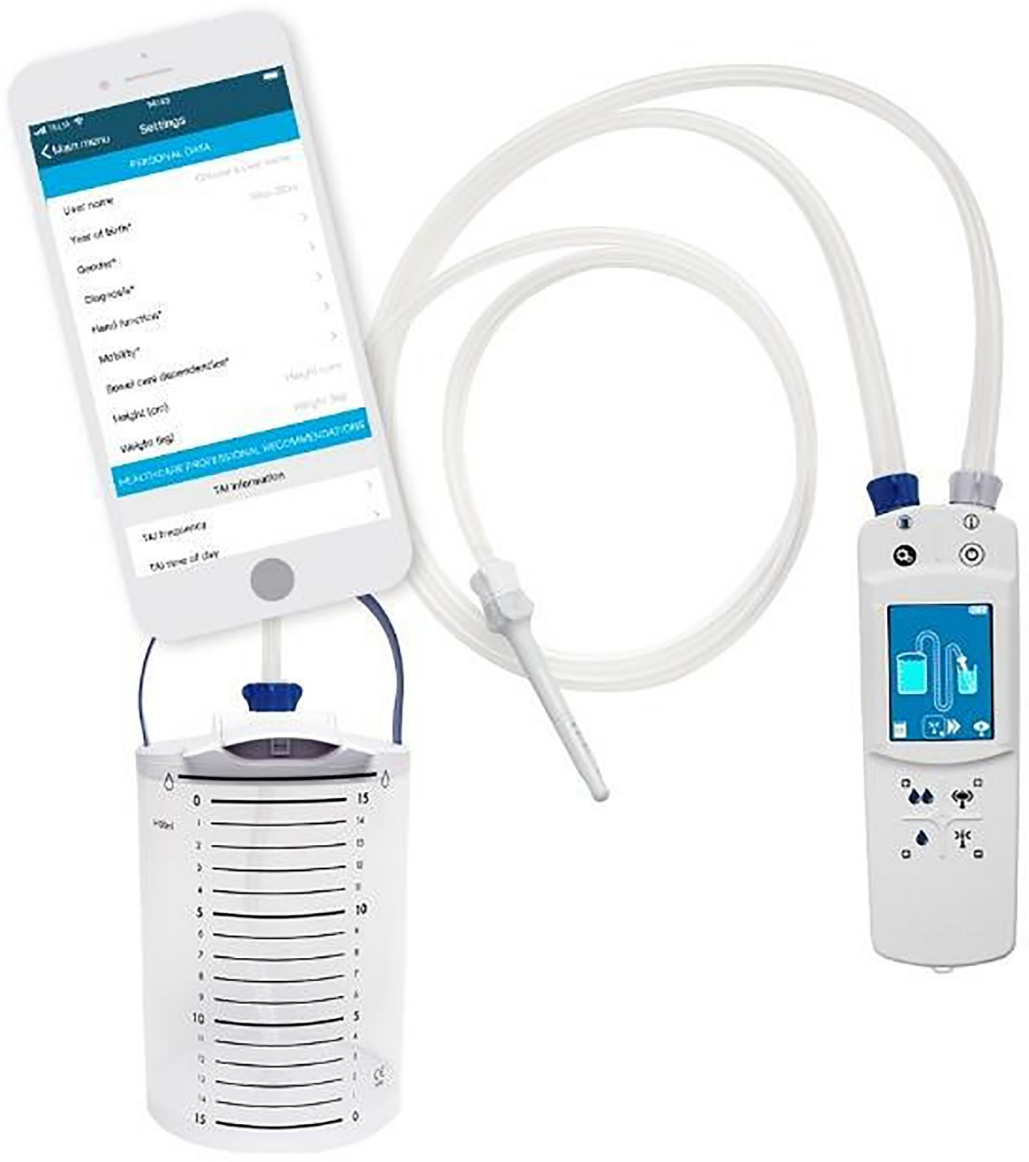
Navina™ Smart system. The Navina Smart system consists of a hydrophilic rectal catheter with an in-/deflatable balloon for retention, tubing, connectors, a water container, an electrical control unit and a smartphone/tablet app.

All subjects were trained in the device, Fig 4, before study start and all, but one, considered the training to be adequate and enough time spent before starting the study. It was therefore not possible to correlate training on study device with satisfaction or NBD score.

The Navina Smart App was used by 90% (47 of 52) of the subjects in the PP data set and 65% (34 subjects) thought it was very easy or easy to synchronize the app with the Navina smart electronic control unit. More than half of the subjects (point estimate 54%; 28 of 52, 95% CI 39% - 68%) considered that the Navina Smart app made them feel in control since they got continuous feedback.

### Safety observations

**Fig 5:**
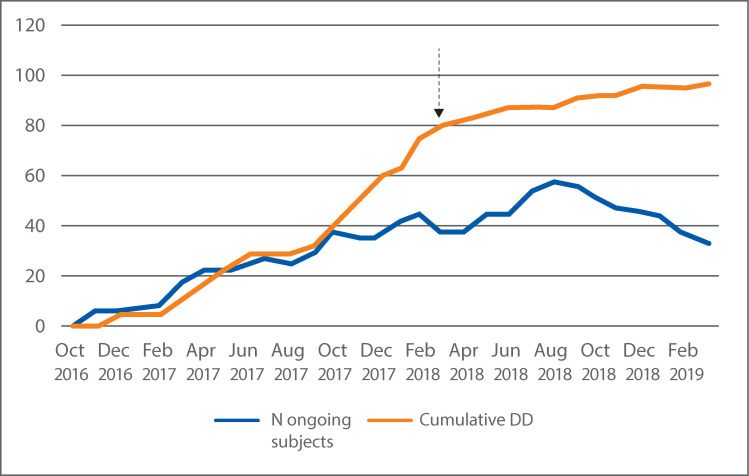
Cumulative Device Defiencies (DD) versus ongoing subjects. The number of DD reports that occurred monthly added together with the number of ongoing subjects each month shows that the first version of the electronic control unit used in the study resulted in most DD reports while continuous product improvements and software updates once the problem was identified and rectified decreased the DD reports (Feb/March 2018 indicated by the arrow). None of the DD resulted in a safety event.

Side effects, collected as adverse events (AEs), that were judged as at least possibly related to the device or therapy were reported in 11 (13%) of the 84 subjects that used the device (Table 3). Of the reported adverse events, one was a serious adverse event, possibly related to the device or therapy. This was a subject who experienced severe autonomic dysreflexia at the first training with the device. The total number of reported device deficiencies (DD) were 97. The most commonly reported DD were failure of the electronic control unit (78%) followed by the rectal catheter (22%). The main failures of the electronic control unit were failure to start and the synchronization with the app i.e. the Blutooth connection. The majority of the device deficiencies (56%) occurred during the subjects´ first 3 months participation in the study. The first version of the electronic control unit used in the study was responsible for most of the DD reports. Continuous product improvements and software updates in line with standard procedures decreased the DD reporting, see Fig 5. None of the DD were reported to result in a safety event.

**Table 3:** Adverse events. Description of possible related / related adverse events that occurred during the study in 11 of 84 subjects. There was one related serious adverse event reported that was a subject who experienced severe autonomic dysreflexia during the first TAI session.

Adverse event per subject (N = 84)	Related to therapy	Serious Adverse Event (SAE)
Failure to empty bowel	Related	No
Dropped stool after irrigation	Possibly related	No
Spasticity increase and bowel incontinence	Possibly related	No
Sweating	Related	No
Sweating	Related	No
Sweating	Related	No
High blood pressure/autonomic dysreflexia	Related	Yes
Leg's spasms	Possibly related	No
Abdominal swelling	Possibly related	No
Minimal anal bleeding and minimal anal pain	Possibly related	No
Abdominal pain after irrigation	Possibly related	No

## Discussion

We present data on the short-term efficacy of Navina Smart in individuals with NBD secondary to spinal cord injury. The study cohort was recruited from SCI centres around Europe and tended to have severe NBD symptoms: 79% had severe symptoms at study entry, which fell to 31% after three months of use of Navina. Symptom burden was assessed by the NBD score, a standard and validated instrument for assessing bowel dysfunction in this cohort. It was therefore unsurprising that the improvement in NBD scores correlated well with subjective satisfaction with bowel function, which rose by a mean of approximately 2 points on a 5-point scale (from 1.85 at baseline to 3.67 at three months).

There was a reduction of mean NBD score by 7.9 (from 17.8 to 9.9) for all subjects, and of 8.5 (from 19.4 to 10.9) for subjects with severe NBD symptoms. This compares favourably with the definitive study of TAI with the Peristeen system that showed a reduction of mean NBD score of 4.4 (from 14.8 to 10.4) [9]. The corollary of this was that dissatisfaction rates were low at 6%, 3 of 52 subjects. In the original study by Christensen et al, discontinuation rates were 12%, with 5 of 42 subjects ceasing therapy. This improvement with Navina was seen in subjects with severe NBD who represented the majority of those recruited to this study.

In addition to the NBD score, there was also an improvement in time needed for the subject’s daily bowel management. The mean time spent on bowel management at baseline was 74 minutes per day, which was reduced by nearly 30 minutes with treatment. The number of steps required for bowel care reduced from a mean of 4 to 3 after treatment, and the steps most subjects no longer needed were digital removal of faeces, digital rectal stimulation and use of rectal suppository. There was a non-significant trend to reduction in pad usage in subjects with faecal incontinence. Minimising incontinence and reducing complexity of bowel management are amongst the key factors that individuals with NBD aim for in managing their condition, based on a discrete choice study [14]. In the patient preference study “risk of faecal incontinence (FI)”, “frequency of use” and “avoiding urinary tract infections (UTIs)” were regarded as the most important features of a TAI device.

As well as being effective and well tolerated, the short-term outcomes showed that Navina was safe to use. Eleven of 84 subjects (13%) reported an adverse event during the 3 months, of which one was an episode of severe autonomic dysreflexia (AD). AD is a common complication of SCI and symptoms include headache, bradycardia, cardiac arrythmias. Severe episodes may be life threatening. However, TAI was not associated with increased risk of AD for individual with SCI compared to digital anorectal stimulation [15]. The other adverse effects were minor, and felt by the investigators to not necessarily relate to the irrigation. The incidence of adverse effects compares with that reported in a long-term cost efficacy study of 28% annually [8]. Long-term use of TAI has been shown to be safe and without any side effects in more than half of the studied population [16]. In a previous study of the Navina system in individuals either intolerant or unresponsive to alternative irrigation regimes, three of 30 subjects had an adverse effect (10%) [12], comparable to the figures in this study. None of these adverse events were thought to be related to the irrigation. All subjects in this study were naïve to TAI and considered that they received adequate training in the study device before starting the study. Training in TAI is important to minimise the risk of complications. A rare complication that is more common in treatment naïve individuals is bowel perforation. Bowel perforations are as rare as 2–6 bowel perforations per million procedures of TAI. Most incidences are reported during the first 8 weeks use of TAI [17].

The chief challenge of this study was the frequency of device deficiencies. The Navina Smart device is a recent development, and technologically a step-up from other devices for TAI in being able to monitor and electronically regulate the irrigation. Despite thorough prior laboratory testing, some minor software deficiencies related to use only become apparent in the actual clinical setting. Consequently, earlier versions of the device were found to have some technical issues that were corrected in subsequent and current versions. The first version of the device used in the study appeared to have bugs in the software resulting in failure of the electronic control units to initiate at the first use of the device. Another bug was with the Bluetooth connection between the electronic control unit and the smartphone/tablet, which made synchronization with the Navina Smart app not possible. The software and device were updated continuously during the study and subjects switched devices after updates. The issues with the devices affected the recruitment of the subjects and therefore, the number of included subjects did not correspond to the 90% power used for determining sample size. However, the number of included subjects in the per protocol analysis set used here are well above the need for detection of a minimum difference of 0.9 in NBD score with 80% power and standard deviation of 2.2. Subjects not willing to continue may also have been affected by the number of device deficiencies, with one subject reporting the reason for not continuing the study being device deficiency, while 10 reported not being willing to continue study for personal reasons. Some of those not willing to continue study had experienced device deficiencies. However, other studies on TAI have discontinuation rates between 45–55% [11, 18], and there may be other reasons for subjects not willing to continue the study.

In a previous study of the Navina system we showed that the system is easily learnt, well tolerated and effective [12]. This study has corroborated the above findings in a larger cohort of subjects. This analysis was a short-term report of outcome(s) from a longer term study which will report findings after 12 months. That full data set would represent the longest large study of prospectively recruited subjects.

Transanal irrigation has become a key component of bowel management in individuals with NBD. Implementation of TAI is suggested after failure of standard bowel care and before more invasive treatments such as sacral nerve stimulation, antegrade colonic irrigation and stoma [19]. A study into the long-term results of TAI found that at long-term follow-up TAI resulted in lower rates of stoma surgery, UTIs, episodes of faecal incontinence with improved quality of life adjusted years compared to standard bowel care [8, 20]. This was associated with cost savings of £21,768 per patient compared to continuation of standard bowel care. TAI has been shown to be beneficial both for the individual with regard to quality of life and the healthcare system as cost-effective [8]. However, 40% of individuals discontinue irrigation, and device development may improve adherence to therapy. The Navina Smart system incorporates an electronic control unit which permits irrigation with the push of a button without the need for hand strength and coordination to control the flow of air and water. The potential advantage of such device improvements over the classical hand-pump system needs formal study, and we have previously shown that Navina allows “salvage” of over 50% of subjects who had failed other irrigation systems [12]. This study of TAI naïve subjects shows adherence with Navina in 73% of subjects after 3 months (61 subjects (intention to treat (ITT) data set) of 84 included subjects).

## Conclusion

From this first efficacy study on Navina Smart we conclude that the system is an effective treatment option for individuals with NBD during the initial three months. The largest effect, in terms of decreased NBD score, was seen in subjects with severe NBD symptoms where more than half of the subjects improved from severe NBD to very minor, minor or moderate NBD. Whilst there were some device related problems, the Navina Smart was well tolerated with only 13% of subjects reporting adverse effects. Twelve months efficacy results will follow, including results on quality of life and patient-perception of Navina Smart.

## Supporting information

S1 TREND checklist (PDF)

S1 Protocol Clinical study protocol for NAV-0001 3.0. (PDF)

S1 Appendix Navina Smart treatment schedule. Introduction to transanal irrigation (TAI) is a highly individual process. The Navina^™^ Smart system settings will benefit the tailoring of the therapy to fit individual needs. This treatment schedule indicates what steps to take when making modifications of the study subject’s irrigation therapy.
(PDF)

S1 Questionnaire The study-specific subject-reported questionnaire used for the 3 months follow up. (PDF)

S1 Data Primary objective data. (XLSX)

S1 File (DOCX)
