# Supplementary material for: An open prospective study on the efficacy of Navina Smart, an electronic system for transanal irrigation, in neurogenic bowel dysfunction
Source: PLoS One. 2021 Jan 29;16(1):e0245453. doi: 10.1371/journal.pone.0245453 (PMC7845961; doi:10.1371/journal.pone.0245453)
Supplement: S1 Protocol — (PDF) [file pone.0245453.s002.pdf]

Study Product:	Navina™ Smart system
Study Code:	NAV-0001
Version No.:	3.0
Version Date:	20 June 2017

AN OPEN, QUALITATIVE, PROSPECTIVE, MULTICENTER TRIAL OF A NOVEL TRANSANAL IRRIGATION SYSTEM IN SPINAL CORD INJURED PATIENTS

Sponsor:
Wellspect HealthCare, Aminogatan 1, SE-432 21 Mölndal, Sweden.

The following Amendment(s) and Administrative Changes have been made to this plan since the date of preparation:

Amendment No.	Date of Amendment	Local Amendment No.	Date of Local Amendment
1.0	14 December 2016		
2.0	20 June 2017		

Administrative Change No.	Date of Administrative Change	Local Administrative Change No.	Date of Local Administrative Change
1.0	10 May 2016		

Wellspect HEALTHCARE

SYNOPSIS

**Study Timetable**	
Number of subjects planned	150
Estimated date of first subject enrolled	October 2016
Estimated date of last subject completed	September 2019

Objectives

The overall objective of the study is to evaluate change in neurogenic bowel dysfunction (NBD) symptoms after use of the Navina™ Smart system. The assumption is that NBD symptoms are constant over time (i.e. 12 months of study duration) and the hypothesis is that any change should be because of the use of the Navina Smart system.

Study Design

This study is designed as an open, prospective, non-controlled, qualitative, multicentre study of a novel transanal irrigation system performed in a population of 150 subjects suffering from spinal cord injury and confirmed neurological bowel dysfunction. The study is expected to last for a total of 1 year (treatment period) with a planned 12- month recruitment period and three scheduled site visits.

Target Subject Population

Male and female subjects, 18 years or above, with traumatic or non-traumatic spinal cord injury, at least 3 months post injury and suffering from confirmed neurological bowel dysfunction will be considered.

Subjects will be consecutively screened in both inpatient and outpatient settings according to the inclusion- and exclusion criteria stated in the study protocol. After eligibility has been confirmed and informed consent has been given, subjects will be assigned to transanal irrigation with the Navina Smart system.

Study Device

Navina™ Smart system.

Primary Objective:

The primary objective of this study is to evaluate change in neurogenic bowel dysfunction (NBD) symptoms between baseline and 3 months use of the Navina Smart system as measured by a patient friendly version of the validated instrument NBD Score.

Secondary Objectives:

- To investigate the change of quality of life in the selected patient population.
- To investigate neurogenic bowel dysfunction symptoms.
- To evaluate study product compliance.

- To investigate patient perception and satisfaction of bowel management with transanal irrigation therapy and the use of the Navina Smart system.
- To investigate frequency of urinary tract infections.
- To investigate parameters relevant for health economic modelling, e.g. cost effectiveness analysis and budget impact modelling.

Safety Objectives:

The safety of the investigational device, Navina Smart system will be monitored during the prospective follow-up (at 3, 6, 9 and 12 months) by continuous reporting of adverse events and serious adverse events. In addition, the safety will be followed during the initial weekly telephone follow-up.

TABLE OF CONTENTS PAGE

1 Background ... 8
1.1 Study Rationale ... 13
1.2 Risk/Benefit Assessment ... 13
2 Study Objectives ... 15
2.1 Primary Objective ... 15
2.2 Secondary Objectives ... 15
2.3 Safety Objectives ... 15
3 Study Design ... 16
3.1 Overall study design ... 16
3.2 Study Visits ... 16
3.3 Study Design Rationale ... 22
4 Study Population ... 22
4.1 Subject Screening Log ... 22
4.2 Inclusion Criteria ... 22
4.3 Exclusion Criteria ... 23
4.4 Restrictions ... 24
4.5 Discontinuation of Study Participation ... 24
4.5.1 Criteria for Discontinuation ... 24
4.5.2 Procedures for Discontinuation ... 25
4.6 Assigning Subjects ID ... 25
5 Study Treatments ... 26
5.1 Study Device ... 26
5.1.1 Component list and Supply scheme ... 27
5.1.2 Navina Smart app and compatible mobile devices ... 29
5.1.2.1 Navina Smart app ... 29
5.1.2.2 Compatible mobile devices ... 29
5.2 Procedures ... 30
5.3 Treatment Compliance ... 31
5.4 Labelling ... 31
5.5 Device Accountability ... 31
6 Study Variables: Definitions and Assessments ... 32
6.1 Screening and Demographic Variables ... 32
6.2 Primary and Secondary Outcome Variables ... 32
6.3 Safety Variables ... 34
6.3.1 Adverse Events ... 34
6.3.1.1 Definitions ... 34
6.3.1.2 Adverse Event and Device Deficiency Reporting ... 35
6.4 Patient-Reported Outcomes (PROs) ... 36
6.4.1 The Neurogenic Bowel Dysfunction score ... 36
6.4.2 The EQ-5D-3L ... 37
6.4.3 The “Baseline Questionnaire” ... 37
6.4.4 The “Follow-up Questionnaire” ... 38
7 Statistical Considerations ... 38
7.1 Statistical Evaluation – General Aspects ... 38
7.1.1 Demographics and Other Baseline Characteristics ... 38
7.1.2 Covariates and Prognostic Variables ... 38
7.1.3 Handling of Dropouts and Missing Data ... 38
7.1.4 Multi-Centre ... 39

Wellspect HEALTHCARE

7.2 Description of Analysis Sets 39
7.3 Method of Statistical Analysis in Relation to Objectives 39
7.4 Determination of Sample Size 39
7.5 Statistical Analyses During the Course of the Study 40
8 Data Management 41
8.1 Electronic Data Capture 41
8.1.1 Navina Smart App Data 41
8.2 Validation 42
9 Study Management 42
9.1 Monitoring 42
9.2 Audits and Inspections 43
9.3 Training of participating centers - Staff 43
9.4 Changes to the Protocol 43
9.5 Deviations from the Protocol 44
9.6 Study Agreements 44
9.7 Before Enrolment 44
9.8 Early Termination of the Study 44
9.9 Publication Policy 44
10 Ethics 45
10.1 Ethics Review 45
10.2 Ethical Conduct of the Study 45
10.3 Informed Consent 45
10.4 Subject Data Protection 46
10.5 Insurance 46
11 Study Contacts 47
12 References 48

LIST OF TABLES PAGE

Table 1 Secondary Objectives 15
Table 2 Safety Objectives 15
Table 3 Study Plan 21
Table 4 Component-list Navina Smart 28
Table 5 Suggested supply scheme – Navina Smart 28
Table 6 Outcome Variables 32
Table 7 Study Contacts 47

LIST OF APPENDICES

Appendix A Signatures
Appendix B Investigators and Study Administrative Structure
Appendix C Navina Treatment Schedule
Appendix D Instructions for Use (IFU)
Appendix E NAV-0001 Data Flow

LIST OF ABBREVIATIONS AND DEFINITION OF TERMS

The following abbreviations and special terms are used in this study protocol.

**Abbreviation or special term**	**Explanation**
ACI	Antegrade Colonic Irrigation
AD	Autonomic Dysreflexia
ADE	Adverse Device Effect (see definition in Section 6.3.1.1)
AE	Adverse Event (see definition in Section 6.3.1.1)
ASIA	American Spinal Injury Association
CRF	Case Report Form
CSA	Clinical Study Agreement
CSP	Clinical Study Protocol
EC, Ethics Committee	Synonymous to Institutional Review Board and Independent Ethics Committee
eCRF	Electronic Case Report Form
ECU	Electrical control unit
EDC	Electronic Data Capture
CSP	Clinical Study Protocol
HRQOL	Health related quality of life
ICF	Informed Consent Form
IFU	Instruction For Use
ISF	Investigator’s Study File
ISO	International Organization for Standardization
ITT	Intention To Treat
LMN bowel syndrome	Lower motor neuron bowel syndrome
MACE	Malone Antegrade Continence Enema
MAUDE	Manufacturer and User Facility Device Experience Database
MS	Multiple Sclerosis
NBD	Neurogenic Bowel Dysfunction
pCRF	Paper Case Report Form
PD	Parkinson´s Disease
PI	Principal Investigator; A person responsible for the conduct of a clinical study at a study site. Every study site has a principal investigator.
PRO	Patient Reported Outcome
PP	Per protocol
PVC	Polyvinyl chloride

Abbreviation or special term	Explanation
PVP	Polyvinylpyrrolidone
QALY	Quality Adjusted Life Years
QoL	Quality of Life
SAE	Serious adverse event (see definition in Section 6.3.1.1)
SADE	Serious Adverse Device Effect (see definition in Section 6.3.1.1)
SB	Spina Bifida
SCI	Spinal Cord Injury
SNS	Sacral Nerve Stimulation
TAI	Transanal irrigation
UMN bowel syndrome	Upper Motor Neuron bowel syndrome
UTI	Urinary Tract Infection
VAS	Visual Analogue Scale
Viedoc™	Web based software used for Electronic Data Capture and Data Management
WHC	Wellspect HealthCare

1 BACKGROUND

Digestion is a fundamental bodily function with the purpose to break down and decompose food and liquids into a form suitable for absorption. The digestive tract consists of multiple organs, stretching from the oral cavity (e.g. teeth, tongue, throat etc.) down to the large bowel. The large bowel comprises the final part of the digestive tract and consists of three parts; the colon, the rectum and the anus. The colon is a muscular tube, around 1,5 m long, and is divided into five major parts; *caecum, colon ascendens, colon transversum, colon descendens* and the *rectum*. The rectum is the last 20 cm of the large bowel and is completed by the anus which comprises the last centimetres. The anus is composed of two distinct sphincters, the *internal* and the *external* anal sphincter.

The functions of the colon are to complete the process of digestion, to form stool and expel faeces from the body, involving absorption of water and minerals. The stool is moved in and through the bowel by coordinated contractions of the muscle layers of the colon towards the anus, i.e. *peristalsis*, with the help of the enteric nervous system. The gastro-colic reflex is a reflex response to the introduction of food and/or drink into the stomach, resulting in an increase in muscular activity throughout the gut[1] which can result in movement of stool into the rectum. Extrinsic neurological input to the gut goes via the autonomic nervous system: a parasympathetic input to the ascending and transverse colon, sympathetic innervation that results in decreased motility and tones in the colon and contracts the sphincters.

The *internal* anal sphincter is composed of smooth muscle and is under reflex control. Even in the absence of normal external innervation the internal sphincter will have some tone and contractility. The *external* sphincter is composed of striated muscle and is under both reflex and voluntary control via somatic lower motor neurons in the pudendal nerve from the sacral cord. In the absence of normal external innervation the external sphincter will be areflexic. The intrinsic or enteric nervous system is embedded within the gut wall, as the submucous (Meissner’s) plexus and the mucosal (Auerbach’s) plexus and coordinates gut secretion, blood flow and muscular activity.

Defecation is defined as the final act of digestion, meaning that waste products are expelled from the body via the anus. In humans, defecation occurs within a range of a few times per day to a few times per week. At the start of the defecation cycle, the rectum ampulla acts as a temporary storage facility for the unneeded material. As additional faecal material enters the rectum, the rectal walls expand. A sufficient increase in faecal material in the rectum causes stretch receptors from the nervous system located in the rectal walls to trigger the contraction of rectal muscles, relaxation of the internal anal sphincter and an initial contraction of the skeletal muscle of the external sphincter. The relaxation of the internal anal sphincter causes a signal to be sent to the brain indicating an urge to defecate.

If this urge is not acted upon, the material in the rectum is often returned to the colon by reverse peristalsis where more water is absorbed, thus temporarily reducing pressure and stretching within the rectum. The additional faecal material is stored in the colon until the next mass 'peristaltic' movement of the transverse and descending colon. If defecation is delayed for a prolonged period the faecal matter may harden and autolyze, resulting in constipation.

Once the voluntary signal to defecate is sent back from the brain, the final phase of the cycle begins. The rectum now contracts and shortens in peristaltic waves, thus forcing faecal material out of the rectum and out through the anal canal. The internal and external anal sphincters along

with the puborectalis muscle allow the faeces to be passed by pulling the anus up over the exiting faeces in shortening and contracting actions.

In some diseases or post-injury conditions defecation disorders are more common and pronounced than in the general population, e.g. conditions affecting/involving the central and peripheral nervous system, i.e. *spinal cord injury* (SCI), *multiple sclerosis* (MS), *spina bifida* (SB), severe *Parkinson's disease* (PD), etc. In the literature patients suffering from such conditions, i.e. bowel-related symptoms and manifestations, are usually also referred to as suffering from *neurogenic (neuropathic) bowel dysfunction* (NBD).[2,3]

Neurogenic bowel dysfunction is the term used to describe dysfunction of the colon due to a disrupted peristalsis, precipitating rectal distension or colonic slowing. Symptoms related to NBD are e.g. constipation, disordered defecation and faecal incontinence. The magnitude of these symptoms is determined by the underlying pathology which in turn is dependent on the extent of injury or damage. Individuals who suffer from NBD often have self-esteem issues affecting personal relationships and their social life.[4,5] Further, the severity of NBD often correlates inversely to quality of life (QoL), and by some populations (i.e. SCI) ranked as greater than other aspects of the condition.[6]

The majority of individuals with SCI experience moderate to severe symptoms related to NBD, e.g. faecal incontinence (42%) [7] and constipation (39-77%). [4,5] The severity of NBD is dependent on the type of spinal injury, or more adequately, the level and completeness of the injury. Consequently, the most severe degree of NBD is seen in individuals with a complete lesion of the spinal cord.[8]

The clinical presentation of bowel dysfunction in the SCI population is often divided into two distinct patterns: injuries above the *conus medullaris* results in *upper motor neuron* (UMN) bowel syndrome and injuries at the *conus medullaris* and *cauda equina* results in *lower motor neuron* (LMN) bowel syndrome.[9,10]

UMN bowel syndrome, or hyperreflexic bowel, is characterized by increased colonic wall and anal tones. Voluntary (cortical) control of the external anal sphincter is disrupted and the sphincter remains tight, thereby promoting retention of stool. The nerve connections between the spinal cord and the colon remain intact, and therefore, there is preserved reflex coordination and stool propulsion. The UMN bowel syndrome is typically associated with constipation and faecal retention at least in part due to external anal sphincter activity.[9] Stool evacuation in these individuals occurs by means of reflex activity caused by a stimulus introduced into the rectum, such as an irritant suppository or digital stimulation.

LMN bowel syndrome, or areflexic bowel, is characterized by the loss of centrally-mediated (spinal cord) peristalsis and slow stool propulsion. LMN bowel syndrome is commonly associated with constipation and a significant risk of incontinence due to the atonic external anal sphincter and lack of control over the *levator ani* muscle that causes the lumen of the rectum to open. Completeness of injury also has a significant impact on bowel function in individuals with SCI. Those with an incomplete injury may retain the sensation of rectal fullness and ability to evacuate bowels so no specific bowel program may be required, however, the pathophysiologic mechanisms of faecal incontinence and constipation in subjects with incomplete SCI are similar to subjects with complete SCI and preserved spinal sacral reflexes. [10]

As previously mentioned, NBD is a major physical and psychological problem for individuals with SCI, as changes in bowel motility, sphincter control, coupled with impaired mobility and

hand dexterity result to make bowel management troublesome and a major life-limiting problem. Having this in mind, it is not surprising that improving bowel function alone, or bladder/bowel functions, is rated among the highest priorities among individuals with SCI.[11,12]

Half of all subjects with SCI have moderate to severe NBD symptoms[13], and there is a significant correlation between the severity of NBD and QoL, especially with regards to physical functioning. Bowel dysfunction caused major restrictions in social activities and in the QoL in 39% of persons with SCI. This was regarded as a greater problem than both bladder and sexual dysfunction[14]. There is a correlation between severity of NBD and impairment of QoL.[13,15]

In the treatment of NBD there is not only the affected central or peripheral nervous system that has to be taken into account. Other medical conditions e.g. colonic cancer, inflammatory bowel disease etc., immobility, dietary changes and pharmacological treatments, e.g. opioids and spasmolytics, co-operate in the development and progress of neurogenic bowel dysfunction. Therefore, all these factors need to be considered for each individual patient.[16-18]

Usually, a thorough medical examination to exclude other pathologies and to map out the present degree of neurogenic bowel dysfunction is undertaken. This examination should at least consist of acquisition of a careful medical history, a digital rectal examination, physical and neurological examination, functional assessment and a radiological examination.[17,18] The use of specific disease severity scales, symptom-related scales (e.g. constipation and faecal incontinence) will further guide the healthcare professional to determine the degree of the disordered defecation and to suggest the level of treatment approach.

The available treatment options are usually divided into different levels depending on the complexity and invasiveness of the intervention.[18]

The first step consists of diet and fluid recommendations, e.g. increase of dietary fibre and fluid intake, lifestyle alterations, e.g. increase level of physical activity, oral laxatives and constipating drugs, e.g. bulking agents, stool softeners etc. Even though these basic interventions are widely recognized and utilized in the medical and nursing communities the scientific evidence base is remarkably scarce and conflicting.[17] Abdominal massage has been suggested as an accessible route in the treatment of disordered defecation and one study on 30 MS patients reported significant improvement of constipation symptoms in the group that received abdominal massage compared to the control group. [19] However, this finding needs to be confirmed in studies with larger populations of patients with NBD.

The second step is based on digital rectal stimulation, suppositories and biofeedback. Digital rectal stimulation is a procedure performed by the insertion of a gloved and lubricated finger into the rectum and anal canal, rotating gently with a circular movement. This procedure activates preserved anorectal reflexes and thus increases motility in the anorectum[20] and the left colon.[21]

A rectal suppository is a torpedo-shaped dosage form that constitutes a mild laxative, e.g. bisacodyl, encapsulated by a white/grey greasy base, often cocoa butter or polyethylene glycol. After the transanal insertion they act locally, by a stimulating effect on the colonic wall, with increased peristalsis. The increased peristalsis, together with increased retention of water and electrolytes, result in a stimulated defecation, shorter colonic transit time and looser stool. Other dosage forms for mild laxatives or bowel stimulants are available, e.g. rectal solutions dispensed through a small plastic tube (i.e. micro-enema). The principle mode of action is basically the same as for the suppositories, i.e. an increased retention of water leading to increased peristalsis and defecation reflex.[22]

Biofeedback is the generic term for; a) ‘training’ the external anal sphincter to increase the squeezing pressure and b) to increase the anorectal sensibility. The former is usually achieved by the insertion of a small anal probe connected to a pressure transducer, thus displaying a signal as the patients learn to squeeze. Accordingly, the latter is achieved by the insertion of a rectal balloon that is gradually filled until the patient is aware of its presence in the rectum. These procedures have to be repeated for several weeks until satisfactory results have been achieved.[16] However, due to the pre-requisite of a preserved rectal sensation this treatment option is best suited for patients with less severe symptoms.[17]

Both the first and second steps on the NBD “treatment ladder” are considered as conservative treatments as they are not invasive, or invasive to a very small extent, i.e. suppositories or micro-enemas.

The third step consists of transanal irrigation (TAI), i.e. instillation of fluid (most often water) into the colon via the anus. Other applicable terms for this intervention are, e.g. ‘retrograde colonic irrigation’, ‘colonic washout’, ‘bowel irrigation’ and ‘enema’. Irrigation of the large intestine has been utilized for medical and non-medical purposes for thousands of years, both as a cleansing method as well as treatment for constipation.[23] The accuracy and appropriateness of bowel irrigation have been questioned from time to time during the history, but nowadays it is a well-documented and frequently used intervention worldwide. Colonic enemas have been administered before radiological examinations of the large bowel since the 1920s, both for the purpose of evacuation of faeces (i.e. sodium phosphate enemas) and as a contrast medium (i.e. barium enemas).[24] In the late 1980s Shandling and Gilmour reported excellent results from a study on bowel management in a SB population.[25] One hundred percent of the patients (n=112) reported symptom relief. Shandling and Gilmour were inspired by colostomy patients using a catheter to irrigate their bowel via the stoma and thus achieved a state of ‘pseudo-continence’. The novel ‘Enema Continence Catheter’, a rectal catheter with an inflatable balloon, was developed and the bowel management procedure consisted of scheduled colonic irrigation with saline. This procedure was then adopted by others and during the late 1990s and onwards the use was spread to other populations suffering from neurogenic bowel dysfunction. Interestingly, the basic principles of the ‘Enema Continence Catheter’ are still valid today, even though the techniques for instilling the irrigation fluid into the colon have evolved more rapidly, i.e. gravity dependent, mechanical or electronically operated devices.

The fourth step, is Sacral Nerve Stimulation (SNS) with an electrode placed through its corresponding sacral foramen.[26] *The fifth step*, is Antegrade Colonic Irrigation (ACI), where irrigation fluid is administrated through a stoma. [27-29] *The sixth step* is implantation of a sacral anterior root stimulator[16] and *the seventh step* is permanent stoma or colostomy. [17,30]

Catheters used for transanal irrigation can basically be divided into two different concepts:

- *Cone-shaped colostomy tip catheters*: Primarily used by patients who irrigate their bowel via a stoma. The cone-shape tip is placed over the stoma orifice and held in place by hand. Accordingly, when used for transanal irrigation the catheter tip is placed at the anus and held in place by hand. Irrigation fluid is then installed into the bowel by gravity-, mechanically or electronically induced pressure.
- *Rectal balloon catheters*: As previously described, this type of catheter is based on the ‘enema continence catheter’, originally developed and described by Shandling & Gilmour in 1987. It was further developed, e.g. by adding a hydrophilic surface, and utilized in other populations suffering from neurogenic bowel dysfunction.[31]

Wellspect HEALTHCARE

There is a selection of devices aimed for transanal irrigation commercially available on the market today. Basically, they can be divided by their mode of action, i.e. how the irrigation fluid is instilled into the bowel, either by simple gravity or by manual or electronic pressure build-up.

The market leader on toilet-based transanal irrigation systems is Peristeen®, developed and manufactured by Coloplast A/S (Denmark). The system is based on the aforementioned single-use hydrophilic rectal balloon catheter, a manual control unit, tubing and a water container. The housing of the manual control unit consists of a pump (for air and water pressure build-up), a switch (between air, water and balloon deflation) and tubing connectors. This particular device has been used in several clinical trials on transanal irrigation in various populations why the therapy in general, and the use of the device in particular, are supported by strong scientific evidence.[23,32,33]

The Qufora system by MBH International (Denmark) offers a range of products for transanal irrigation; the Mini system, the Toilet system, the Irrigation system with rectal balloon catheter and the Bed system. The Mini system consists simply of a pump and a rectal cone, thus allowing rectal irrigations only. The Toilet system consists of a water container, tubing, pump and a rectal cone. The system can also be used without the pump, thus relying on gravity-induced pressure. The Irrigation system with rectal balloon catheter shares some similarities to the Peristeen® by Coloplast, but with the exception that the silicone-based catheter is for re-use (up to 3 times) and the balloon is to be filled with water instead of air. The Bed system is for users who are not ambulatory, cannot sit on a toilet seat etc. The system is closed and consists of a water container, tubing, a rectal catheter, collection container and can be used with or without a pump.

The company B.Braun (Germany) has a wide range of devices for irrigation even though they are primarily aimed for colostomy irrigation. One of the devices, the Biotrol® Irrimatic, is an electronic irrigation pump with a cone-shaped catheter tip. This pump has been used in at least two clinical trials of retrograde colonic irrigation (i.e. transanal irrigation) and with favourable outcomes. However, these trials were conducted between 1999 – 2003 [34] and 2003-2004 [35] and according to the manufacturer's web-site, the range of application covers irrigation of colostomies only.

In contrast to this, another electronic pump by B.Braun, Irrimatic R, is marketed as a pump for rectal/intestinal flushing. Among the accessories accounted for, a catheter for rectal use (ReClean®) is displayed. This catheter contains an adjustable cone. It is unknown to date if the electronic units are the same but marketed differently due to the range of application. There are no clinical data publically available for this device (incl. the ReClean® rectal catheter).

The Navina Systems (referred to as either "Navina Systems" in general or "Navina Smart", "Navina Smart system" or "Navina Classic" in particular) for transanal irrigation, developed and assembled/manufactured by Wellspect HealthCare (WHC), consist of two different handheld control units, a water container, tubing, connectors and a rectal balloon catheter. One of the control units, Navina Smart, is electronically powered while the other one, Navina Classic, is manually powered and operated. Navina Systems is developed to help adults who suffer from faecal incontinence, chronic constipation, and/or time consuming bowel management.

Navina Systems can be used at home, away from home, on travel or in health care settings.
The Navina Smart system is the investigational device in the context of this clinical study.

1.1 Study Rationale

This study is undertaken in order to gather clinical data on the use of the newly developed Navina System. The main objective is to evaluate the change in NBD symptoms after 3 months use of Navina Smart. The study will include patients with confirmed NBD refractory to conservative therapy and judged eligible for transanal irrigation.

Since the product is CE-marked based on similarities to existing products it is concluded that there is no need to conduct a study for regulatory purposes and thus a single arm study design has been chosen. However, in order to assure healthcare professionals and users that the product and concept is effective, reliable and safe, it is essential to conduct a sufficiently powered clinical study with clinically relevant endpoints. In addition, the study is intended to assess whether the enhanced functionality of the Navina Smart system, in terms of flexibility in tailoring irrigation regimes, is associated with enhanced outcomes and greater patient tolerability. Further, from a post-market surveillance perspective it is considered important to conduct this study in order to satisfy possible future ethical and regulatory demands.

In a randomized controlled trial investigating TAI[32], it was observed that the frequency of urinary tract infections (UTIs) with prescribed antibiotics were significantly lower in the group using TAI (5.9%) compared to the group using conservative therapy (15.5%). The mechanism in the interaction between bladder and bowel dysfunction is complex and not yet understood. This study will further investigate these findings.

1.2 Risk/Benefit Assessment

There are several side effects or problems reported with the use of TAI, though most of them are mild and of minor concern. These complications should be viewed in the light of complications related to NBD, such as severe constipation, faecal incontinence and/or bowel-related surgical procedures.

Rectal perforation is considered the most serious side effect and the estimated incidence (from 2009) of rectal perforation due to TAI is 0,002% (one per 50 000 procedures) [36]. However, the latest data on perforation indicates a decrease to 0.0002% (one per 500 000 procedures) [37]. This may be compared to the risk for rectal perforation due to colonoscopy; 0,1% (one per 1000 procedures) [38,39] and flexible sigmoidoscopy; 0,0025% (one per 40 000 procedures)[40] respectively.

In the context of TAI with a rectal balloon catheter, perforation of the rectum may occur due to 3 major causes;

- Direct impaling trauma
- Over-inflation of the balloon
- Exaggerated hydrostatic pressure during water instillation

Perforations due to direct impaling trauma will most often be located in the rectum or in the anal canal. If the perforation affects only the extra-peritoneal perirectal space it is possible that the event will be asymptomatic and stay unrecognized. It is possible that this event may not necessitate surgery, but drainage and antibiotics instead. Should however, the intra-peritoneal space be affected, it is considered as an acute medical emergency condition that most often requires abdominal surgery with the formation of a stoma[18].

At the ISCOS meeting in October 2013 the company Coloplast A/S (Denmark) held a symposium regarding bowel perforations due to the use of TAI with their device Peristeen®. They reported 46 rectal perforations since the introduction of the product, and with a sudden increase of events in 2010 and 2011. After some alterations in catheter design (e.g. catheter tip, balloon) and properties (e.g. stiffness and flexibility) the event rate decreased, according to Coloplast, the following years. From the presented data it is evident that the vast majority of the perforations occur in the rectum (~ 46%), that there is almost an equal distribution between sub- and intra-peritoneal relation, and that almost 70% of the cases are treated with the formation of a stoma. Out of the 46 reported cases, the outcome was fatal in three of them (6.5%). The overall experience gathered by Coloplast led them to state that approximately 60% of perforations will occur within the first five weeks of use and that the perforation rate is not cumulative (i.e. increasing over time). Prior surgery to or disease in the pelvic area are major contributing factors.[41]

Over-inflation of the balloon and/or bursting of the balloon are often mentioned as potential causes of intestinal perforation when performing transanal irrigation. However, it is just occasionally mentioned as the single causative factor when reviewing incident reports in applicable public databases. One of these databases is the ‘Manufacturer and User Facility Device Experience Database’ (MAUDE), a passive surveillance database hosted by FDA. Out of 43 incident reports between 1st January 2011 to 31st December 2015 for Peristeen, four reports involves a burst balloon as the specific or likely cause of intestinal perforation and in a few more cases it is mentioned that the user experienced problems due to a burst balloon, even though not resulting in a perforated bowel [42]. However, there are probably a large number of unreported cases, especially when considering data reported by Christensen and co-workers; one out of three Peristeen users experienced balloon burst during irrigation.[33]

Autonomic dysreflexia (AD) is a potentially life-threatening acute condition requiring immediate emergency care. AD most typically occurs in the SCI population, especially those with a lesion at or above the T6 spinal cord level[43]. Acute AD is a reaction of the autonomic nervous system to overstimulation and is characterized by paroxysmal hypertension with throbbing headaches, profuse sweating, nasal stuffiness, flushing of the skin above the level of the lesion, slow heart rate, anxiety, and sometimes by cognitive impairment[44]. AD is believed to be triggered by afferent stimuli (nerve signals that send messages back to the spinal cord and brain) which originate below the level of the spinal cord lesion. The most common causes of autonomic hyperreflexia seen in patients with SCI are loss of bowel and bladder function, resulting in impaction in the case of the bowels and distention in case of the bladder. Proper treatment of AD involves administration of anti-hypertensives along with immediate determination and removal of the triggering stimuli. The rectum should be cleared of stool impaction, using lubricating gel. If the noxious precipitating trigger cannot be identified, drug treatment is needed to decrease elevating intracranial pressure until further examination can identify the cause.

In the only randomized controlled study to date, investigating TAI, symptoms related to AD tended to be more common in the non-TAI group [32] , potentially suggesting that TAI may have a protective effect against AD. [23]

Additional difficulties [18] that may arise during irrigation are e.g.; bleeding, pain, autonomic symptoms (sweating, palpitations and dizziness), leakage of water around the catheter/cone, difficulties inserting catheter/cone or instilling irrigant, retention of the instilled irrigant, no evacuation of stool after the TAI procedure, faecal incontinence and/or leakage of irrigant between TAI procedures.

A TAI procedure should always be carried out with caution. Bowel perforation is an extremely rare, but serious and potentially lethal complication to TAI, and will require immediate admission to hospital. A patient must contact the study investigator immediately if, during or after anal irrigation, he or she experiences any of the following:

- Severe or sustained abdominal pain or back pain, especially if combined with fever.
- Severe or sustained anal bleeding.

2 STUDY OBJECTIVES

2.1 Primary Objective

The primary objective of this study is to evaluate change in neurogenic bowel dysfunction symptoms between baseline and 3 months use of the Navina™ Smart system, as measured by a patient friendly version of the validated instrument NBD Score.

2.2 Secondary Objectives

The secondary objectives and outcome variables are shown in the table below:

Table 1 Secondary Objectives

Secondary Objective	Secondary Outcome Variable
To investigate the change of QoL status in the selected patient population (absolute values)	EQ-5D-3L variables
To investigate NBD symptoms after 6, 9 and 12 months use of Navina Smart system	NBD score
To investigate study product compliance	PRO variables ("Do you still perform TAI with Navina Smart?" Yes/No)
To investigate patient perception and satisfaction of bowel management including TAI therapy and use of the Navina Smart system	PRO variables
To investigate frequency of UTI	PRO variables
To perform health economic analyses using QoL data and PRO variables	EQ-5D<br>PRO variables

2.3 Safety Objectives

The safety objectives and variables are shown in the table below:

Table 2 Safety Objectives

Safety Objective	Safety Variables
To evaluate short- and long-term safety of the product and therapy	Adverse events, serious adverse events, adverse device effects, etc.

Wellspect HEALTHCARE

3 STUDY DESIGN

3.1 Overall study design

This Clinical Study Protocol has been subjected to a peer review according to WHC standard procedures.

This study is designed as an open, prospective, non-controlled, qualitative, multicentre study of a novel system for TAI performed in a population of 150 subjects suffering from spinal cord injury, both traumatic and non-traumatic, and confirmed NBD. The main objective is to evaluate the change in NBD symptoms between baseline (before treatment start) and 3 months use of Navina Smart system.

Secondary objectives are to investigate the change of quality of life between baseline (before treatment start) and 3 and 12 months use of Navina Smart respectively, to evaluate improvements regarding constipation, faecal incontinence and frequency of patient reported UTIs, evaluate system compliance, and reasons for non-compliance, perception/satisfaction of the product and therapy as well as short- and long-term efficacy and safety at each follow-up; 3, 6, 9 and 12 months, respectively.

The study is expected to last for a total of 1 year for each subject, with a planned 12 months recruitment period and three scheduled site visits (Baseline, 3 months and 12 months). However, as the yearly incidence of subjects who are considered for TAI treatment is somewhat uncertain, it is recognized that the recruitment period may be prolonged until a sufficient number of evaluable subjects have been reached.

Data will be entered into electronic Case Report Forms (eCRFs) using Viedoc, a web based EDC system. At each irrigation episode, data will be stored in the Navina Smart system unit. The subject will transfer this data to the investigator via a compatible smartphone or tablet using the Navina Smart app.

The patient population
Spinal Cord Injury (SCI) may be traumatic or non-traumatic including infection, inflammation, compromised blood supply, post-ischemic etc., and may be referred to as a spinal cord lesion. The abbreviated term SCI will be used throughout the protocol to represent spinal cord damage of any aetiology. Approximately 150 male and female subjects following at least 3 months after the injury, treatment-naïve to TAI, 18 years and above and suffering from confirmed NBD will be consecutively screened and assigned to TAI with the Navina Smart system. The planned number of subjects will be recruited from 10-12 SCI centres in approximately 9 European countries. Subjects will be screened prospectively in both inpatient and outpatient settings according to the inclusion- and exclusion criteria stated in sections **4.2** and **4.3** of this protocol.

3.2 Study Visits

Visit 1 – Baseline

After explaining the study purpose, risks and benefits to the potential subject, the investigator shall allow the subject time to carefully consider participation in the study and to ask any questions about it. After obtaining the subject’s signed Informed Consent and verifying that the

subject fulfils all inclusion and none of the exclusion criteria, the subject will be enrolled in the study and allocated a Subject ID in a consecutive order.

NB: In the presence of a symptomatic UTI a potential subject *should not be enrolled* (see exclusion criteria no 10). After successful treatment of a UTI, the subject may be rescheduled for visit 1 in order to re-evaluate study participation.

The original ICF will be stored in the Investigator's Study File (ISF) and a copy will be given to the patient.

At Visit 1, study **site personnel** will:

- Collect baseline demographics data as well as medical and surgical history and register in the eCRF.

- Document extent and severity of the spinal cord injury according to the American Spinal Injury Association (ASIA), as ASIA score A, B, C, or D in the eCRF.

- Provide subject with instruments for the collection of primary and secondary endpoints to be completed by the subject:

    - NBD score, **See section 6.4.1**
    - EQ-5D-3L, **See section 6.4.2**
    - Baseline Questionnaire, **See section 6.4.3**

- Train the subject in TAI management as a concept and in the Navina Smart system in particular. **See section 5.2**

- Dispense study products according to the "Component list and Supply scheme". **See section 5.1.1**

- Make sure the subject has a compatible mobile device with the Navina Smart app installed and configured. **See section 5.1.2**

- Schedule a telephone contact one week after Visit 1.

- Schedule visit 2 (3 months follow-up).

- Update the subject's medical records with information about study participation, including the study code, a brief description of the study and the date subject signed the ICF.

Weekly Telephone follow-up

- Investigator or delegate will make a telephone call to the subject once per week until a feasible and satisfactory routine has been established, as agreed by both subject and investigator or delegate. The follow-up phone calls will be initiated by the therapist with the intention of identifying optimal performance and outcomes of irrigation, based on irrigation reports sent to site by the subject. If there are specific problems reported by the patient, or there is inadequate efficacy of treatment the therapist will advise tailoring of the TAI regime according to the Navina Treatment Schedule (see Appendix C). The health

Wellspect HEALTHCARE

status and compliance to the therapy will be monitored and collection of any AE and SAE will be performed.

Visit 2 – 3 months follow-up

<u>At Visit 2, study **site personnel** will:</u>

- Provide subject with instruments for the collection of primary and secondary endpoints to be completed by the subject:
  - NBD score, **See section 6.4.1**
  - EQ-5D-3L, **See section 6.4.2**
  - Follow-up Questionnaire, **See section 6.4.4**
- Document any AE/SAE and any changes in additional medications in the eCRF.
- Dispense study products according to the "Component list and Supply scheme". **See section 5.1.1**
- Schedule the 6 and 9 months telephone follow-up.

Telephone follow-up

- Investigator or delegate will make a telephone call to the subject at 6 and 9 months follow-up. The follow-up phone calls will be initiated by the therapist with the intention of confirming optimal performance and outcomes of irrigation, based on irrigation reports sent to site by the subject. If there are specific problems reported by the patient, or there is inadequate efficacy of treatment the therapist will advise tailoring of the TAI regime according to the Navina Treatment Schedule (see Appendix C). The health status and compliance to the therapy will be monitored and collection of any AE and SAE will be performed.
- Schedule visit 3 (12 months follow-up).

Visit 3 – 12 months follow-up and Study Termination

<u>At Visit 3, study **subject** will:</u>

- Return remaining study products.
- Return the borrowed Smartphone (if borrowed at baseline).

<u>At Visit 3, study **site personnel** will:</u>

- Provide subject with instruments for the collection of secondary end points to be completed by the subject:
  - NBD score, **See section 6.4.1**
  - EQ-5D-3L, **See section 6.4.2**
  - Follow-up Questionnaire, **See section 6.4.4**

- Document any AE/SAE and/or changes in additional medications in the eCRF.

- When all questionnaires have been completed and all data entered into the study database, terminate the subject from the study in eCRF (Viedoc).

Study Flowchart

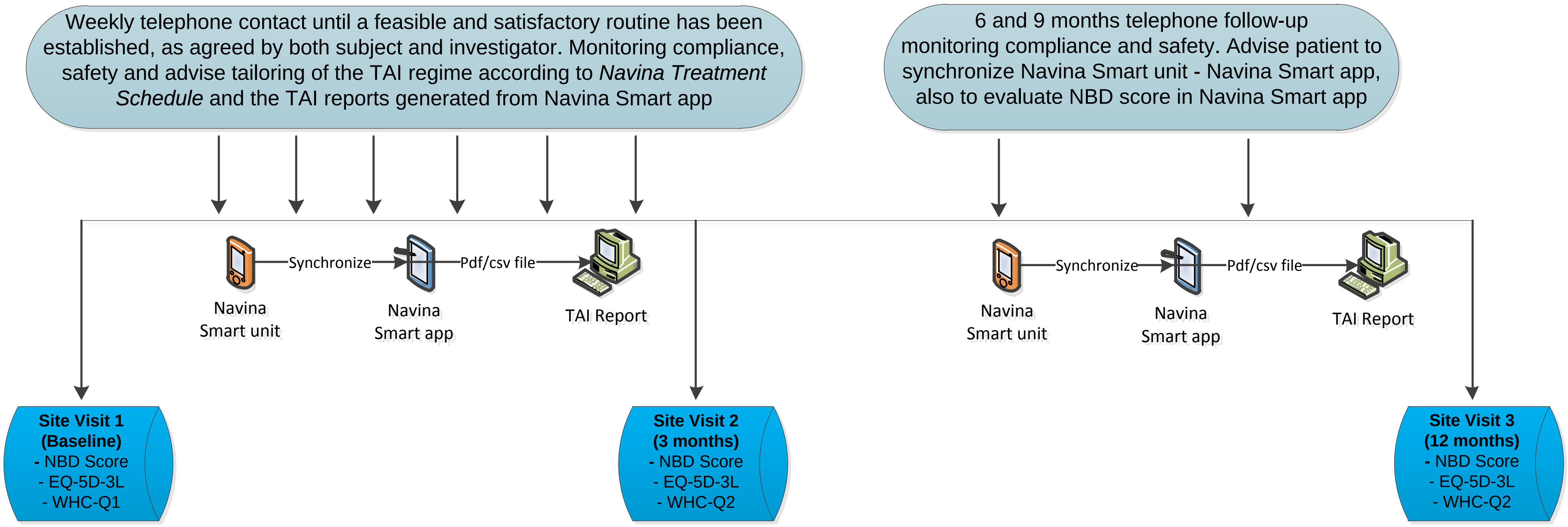

Table 3 Study Plan

	Visit 1<br>Baseline	Weekly telephone follow-up	Visit 2<br>3 months follow-up	6 months telephone follow-up	9 months telephone follow-up	Visit 3<br>12 months follow-up and Termination
**Visit Window**			(± 2 w)	(± 2 w)	(± 2 w)	(± 2 w)
Informed Consent and Subject demographics	X					
Medical/Surgical history	X					
Inclusion/Exclusion criteria	X					
Disease specific severity score	X [a]					
NBD symptoms	X [b]		X [b]	X [b]	X [b]	X [b]
Training/ Education on Navina Smart	X					
Product dispensation	X		X	X	X	
Telephone follow-up		X [c]		X [d]	X [d]	
Smartphone dispensation	X [e]					
AE/ADE/SAE/SADE	X		X	X	X	X
Quality of Life [f]	X		X			X
Evaluating current bowel care	X [g]					
Evaluating Navina Smart system and TAI therapy			X [h]			X [h]

[a] ASIA scale,
[b] NBD score
[c] Weekly telephone call until patient has an established routine
[d] Telephone Follow-up at 6 and 9 month use of Navina Smart
[e] Bluetooth Low Energy protocol (also called Bluetooth Smart or Version 4.0+ of the Bluetooth)
[f] EQ-5D-3L
[g] Baseline Questionnaire
[h] Follow-up Questionnaire

3.3 Study Design Rationale

Transanal irrigation of the bowel can be defined as a process of facilitating evacuation of faeces from the rectum and descending colon by passing water into the bowel via the anus. Two recent reviews[3,20] have suggested that in individuals with chronic NBD, irrigation outperforms more conservative methods, reducing faecal incontinence and constipation, and as a consequence also improves self-reported quality of life. However there is currently lack of evidence identifying robust criteria for suitability, the impact of long term use on colonic function and reason for poor compliance over time. Thus, we have designed an open, prospective, non-controlled, qualitative multicentre study with the primary objective to evaluate the change in NBD score after use of the novel Navina Smart system. The assumption is that the NBD symptoms are constant over time (i.e. 12 months of study duration) if not treated in any way. The hypothesis in the present study is that any change in NBD Score is because of use of the Navina Smart system and hence no control group is required.

Secondary objectives are e.g. to evaluate compliance (and reasons for non-compliance) to the therapy and study product; change in QoL and patient-reported efficacy and safety. In addition, the study is intended to assess whether the use of Navina Smart unit and Navina Smart app, in terms of flexibility in tailoring irrigation regimes, is potentially associated with enhanced outcomes and greater patient tolerability. By synchronizing the Navina Smart unit with the Navina Smart app, a number of irrigation related variables (e.g. balloon size, water amount, instillation flow rate etc.) will be extracted and e-mailed by the subject as reports to the investigator or other designated study personal. This is to empower the patients in their treatments and illuminate the potentially enhanced outcomes and greater patient tolerability.
Due to the nature of the therapy itself, it is not regarded as possible to adopt a blinded design approach. Since the product is CE-marked, based on similarities to existing products and concept, a single arm study design with each study subject acting as its own control has been chosen. Within the population suffering from SCI and a concurrent NBD there is a relatively low incidence of individuals considered for TAI and therefore a multicentre approach is deemed necessary. Otherwise there is a potentially high risk for a prolonged recruitment period.

4 STUDY POPULATION

4.1 Subject Screening Log

The investigator must keep a record of subjects who were considered for enrolment but were never enrolled, e.g., a subject screening log. This information is necessary to establish that the subject population was selected without bias.

4.2 Inclusion Criteria

For inclusion in the study subjects must fulfil all of the following criteria:

1. Provision of informed consent.

2. Male or female aged 18 years or older.

3. Patient with previously confirmed chronic spinal cord injury, **either:**

a) Traumatic; at any level and any completeness of injury and ASIA grade classification,

or

b) Non-traumatic; due to infection, inflammation (i.e.; transverse myelitis or ischaemic myelitis), compromised blood supply, a post-ischemic state, etc.

4. At least 3 months post spinal cord injury at time of consent.

5. NBD score ≥10, confirmed at Baseline[a].

6. Only TAI treatment - naïve patient (not having previously used any particular transanal irrigation system, e.g. Peristeen®).

7. Confirmed NBD refractory to conservative therapy and judged eligible for transanal irrigation as per standardised treatment pathway [18].

8. Able to handle smartphone/tablet.

4.3 Exclusion Criteria

Any of the following is regarded as a criterion for exclusion from the study:

1. Any confirmed or suspected diagnosis of anal or colorectal stenosis, active inflammatory bowel disease, acute diverticulitis, severe diverticulosis, colorectal cancer, ischemic colitis, history of life-threatening autonomic dysreflexia, bleeding disorders, unspecified peri-anal conditions.

2. Untreated rectal impaction.

3. Any radiotherapy to the pelvis.

4. Any current treatment with anticoagulants (not including aspirin or clopidogrel).

5. Any current treatment with long-term systemic steroid medication (not including inhalation agents and/or local topical treatment).

6. Current use of prokinetics.

7. Any prior rectal or colorectal surgery within 3 months

8. Anal, rectal and/or colonic endoscopic polypectomy within the previous 4 weeks.

9. Overt or planned pregnancy.

10. Ongoing symptomatic UTI as judged by investigator.

11. Diagnosed psychiatric illness, considered as unstable by the investigator.

[a] An enrolled subject scoring <10 on the Baseline NBD Score should be discontinued from the study.

12. Diagnosed with MS.

13. Involvement in the planning and conduct of the study (applies to both WHC staff and staff at the study site).

14. Previous enrolment in the present study.

15. Simultaneous participation in another clinical study that may interfere with the present study.

4.4 Restrictions

Below restrictions must be applied on all study subjects:

1. No other system for TAI, beside Navina Smart system, may be used during the study period.

2. Each rectal catheter may only be used once.

3. Use of prokinetics is not allowed during study participation.

4. Use of anticoagulants is not allowed during study participation.

5. Use of long-term systemic steroid medication (not including inhalation agents and/or local topical treatment) is not allowed during study participation.

6. Start of opioid (or equivalent) use during the study is prohibited.

7. Stable use (i.e. no change in the last 3 months before enrolment) of laxatives is permitted but should not be altered during the study period.

It is well documented that certain medications can cause or increase faecal incontinence or constipation. Therefore the health care provider/Investigator must review all the medications the patient takes before enrolment into the study and carefully consider if some medications may have the obvious potential to interfere with a successful outcome of the TAI procedure.

4.5 Discontinuation of Study Participation

4.5.1 Criteria for Discontinuation

Subjects may be discontinued from study participation/treatment at any time. Specific reasons for discontinuing a subject from this study are:

- Voluntary discontinuation by the subject who is at any time free to discontinue his/her participation in the study, without prejudice to further treatment.
- Safety reasons as judged by the investigator and/or WHC.
- Severe non-compliance to protocol as judged by the investigator and/or WHC.
- Incorrect enrolment i.e., the subject does not meet the required inclusion/exclusion criteria for the study.
- Subject lost to follow-up.

Handling of data for discontinued subjects is described in **Section 7.2**.

4.5.2 Procedures for Discontinuation

Subjects not meeting the inclusion/exclusion criteria for this study should, under no circumstances, be enrolled into the study. If subjects not meeting the study criteria are enrolled in error, or where subjects subsequently meet the criteria for discontinuation (see Section 4.5.1) after enrolment, they must be discontinued from study participation/treatment immediately. When applicable, subjects who discontinue prematurely, for any reason, should always be asked about the reason(s) for their discontinuation, the presence of any AE, completion of applicable sections of the eCRF and relevant PROs.

To the furthest extent possible, subjects who discontinue should be seen and assessed by an investigator for follow-up of AE and return of study devices and other study materials.

4.6 Assigning Subject ID

Each participating centre will be given a range of Subject ID for use for their included subjects. The distribution between clinics will be as follows:

Centre No.	Subject No. range	Subject ID range
01	01-99	01-01 -- 01-99
02	01-99	02-01 -- 02-99
XY	01-99	XY-01 -- XY-99

When all eligibility criteria are fulfilled and after obtaining the subject’s signed ICF, the subject will be consecutively allocated a subject id (the only identifier used on any study data).
If a subject discontinues the study, the subject number will not be reused, and the subject will not be allowed to re-enter the study.

5 STUDY TREATMENTS

5.1 Study Device

The Navina Smart system is intended for transanal irrigation.

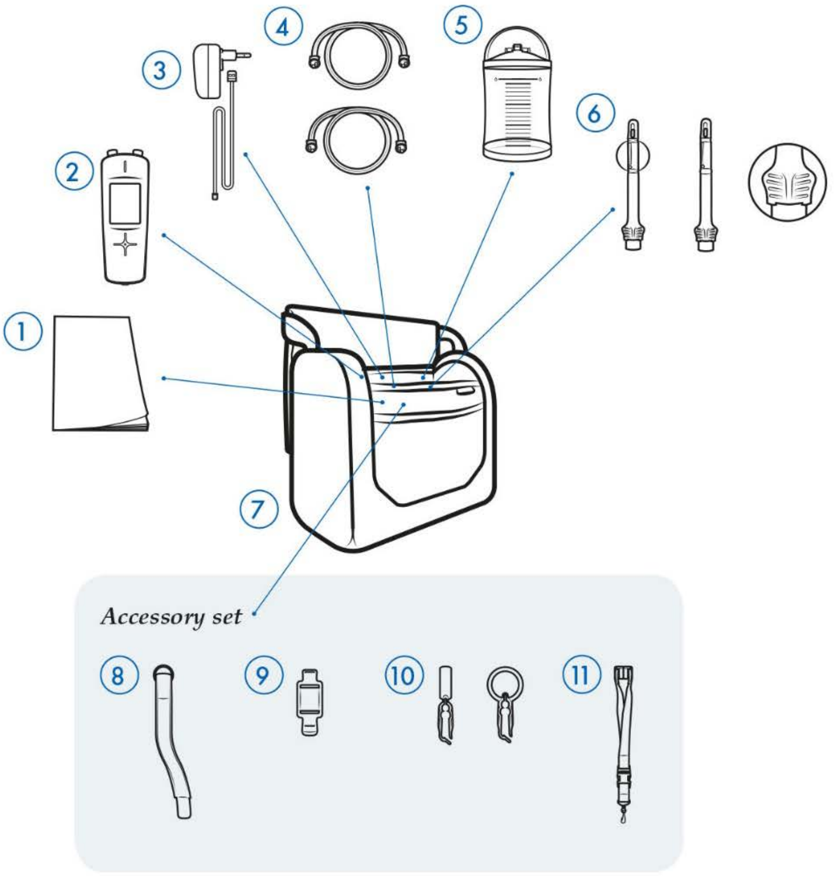

#	Product	#	Product
1.	*Navina Smart instructions for use*	7.	*Navina case*
2.	*Navina Smart control unit (68748)*	8.	*Navina positioning strap*
3.	*Power adapter and cable*	9.	*Navina Smart control unit positioning clip*
4.	*Navina tube set*	10.	*Navina grip rings*
5.	*Navina water container*	11.	*Navina lanyard*
6.	*Navina catheter regular - For adult use only*		

Navina Smart system consists mainly of a hydrophilic rectal catheter with an in-/deflatable balloon for retention, tubing, connectors, a water container and a handheld electrical control unit (ECU). The control unit regulates the pressure to the water container, the water volume as well as inflation and deflation of the balloon on the rectal catheter. The ECU also registers and stores data and settings (balloon size, water volume, water flow rate as well as start and stop time of irrigation) during each irrigation. The catheter is made of Mediprene material which is Polyvinyl chloride and latex free to minimize allergy risks. The material is composed to create a flexible catheter without making it too soft, and the surface has a hydrophilic coating consisting of polyvinylpyrrolidone (PVP). The coating layer binds water and gives a wet and slippery surface that reduces friction against the rectum wall. During instillation of water, the catheter is held in place by an inflatable balloon, also subjected to the hydrophilic coating. The instilled water keeps the catheter and balloon surface moist and slippery throughout the entire treatment.

The hydrophilic rectal catheter is for single-use, the other components may be used multiple times (see “Component-list and supply scheme” below).

Navina Smart is indicated for use by adults with NBD who suffer from faecal incontinence, chronic constipation, and/or time consuming bowel management procedures. By instilling water through the rectal catheter, up into the lower part of the colon, the peristaltic muscles in the bowel can be triggered and start to evacuate the colon and rectum.

At visit 1 the responsible investigator/healthcare provider must train the patient in using the Navina Smart system. Navina Smart can be used at home, away from home, on travel or in a health care setting.

The Navina Smart system will be CE-marked and commercially available prior to study start.

5.1.1 Component list and Supply scheme

The *study product boxes* with system components will be prepared by WHC. During, or after, the irrigation training session the investigator or delegate at the local study clinic is responsible for assigning correct equipment to the subject and also to record the Ref and Lot numbers of the allocated devices in the ”Study Product Accountability Log” in the eCRF. They will also make sure that the subjects receive a complete system and sufficient with associated system components, according to “Component list”. The tables below describe the Navina Smart components and the content of every set that will be supplied (Table 4) as well as a suggested device supply scheme (Table 5). However, it is very important to discuss/agree with the subject how often he/she wants to have system components replenished.

Table 4 Component list – Navina Smart

<table>
<tr><th>Device-kit</th><th>System components</th><th>Lifetime</th></tr>
<tr><td>Navina Smart system regular (“Start kit”)</td><td>• 1 Navina Smart control unit with charger and power adapter
• 1 Water container
• 1 Tube set
• 2 Rectal catheters
• 1 Accessory set
• 1 Navina case</td><td rowspan="4">• Control unit – 400 uses or 2 years from the date of the first use
• Water container - 15 uses
• Tube set - 100 uses
• Rectal catheter - single use</td></tr>
<tr><td>Consumable set</td><td>• 1 Water container
• 15 Rectal catheters</td></tr>
<tr><td>Rectal catheter set</td><td>• 10 Rectal catheters</td></tr>
<tr><td>Tube set</td><td>• 1 Water container tube
• 1 Rectal catheters tube</td></tr>
<tr><td>Accessory set</td><td>• 2 Grip rings
• 1 Lanyard
• 1 Positioning strap
• 1 Navina Smart control unit positioning clip</td><td></td></tr>
</table>

Table 5 Suggested supply scheme – Navina Smart

Visit/Follow up	Device kit to supply	# Rectal catheters regular
Baseline	• Navina Smart system regular (“start kit”)<br>• 2 Consumable set<br>• 3 Rectal catheter set	• 62 catheters in total
3 months follow up	• 3 Consumable set	• 45 catheters in total - enable irrigation every other day
6 months follow up	• 3 Consumable set<br>• 1 Tube set	• 45 catheters in total - enable irrigation every other day
9 months follow up	• 3 Consumable set	• 45 catheters in total - enable irrigation every other day

5.1.2 Navina Smart app and compatible mobile devices

5.1.2.1 Navina Smart app

Wellspect HealthCare is developing an application – the Navina Smart app (the App) – for mobile devices to be used together with the Navina Smart system. The App is developed as a native app, one for iOS and one for Android, distributed for free via AppStore and Google Play and will be CE-marked at time of study start. Usage of the App requires access to a Navina Smart system unit. The App collects irrigation data from the Navina Smart system via a function in the App called "Synchronize". Data is transferred to the App from the ECU via Bluetooth technique. Once stored in the App, the irrigations can be rated as well as analysed in the report module. The App also offers functionality for the user to make bowel management evaluations, e.g. NBD Score, and to generate reports.

The App is intended to help Navina Smart users to fine tune their therapy settings in a timely manner, by sharing their irrigation data with a health care professional. This can be done by sending reports as attachments in an e-mail or by showing the App in a meeting with the healthcare professional.

The App is indicated for users of the Navina Smart system. In this study it is mandatory for the subjects to use the App.

5.1.2.2 Compatible mobile devices

In order to use the App it needs to be downloaded to a compatible mobile device (e.g. smartphone or tablet). Requirements on the mobile device that must be fulfilled are specified below:

- Able to communicate via Bluetooth Low Energy protocol (also called Bluetooth Smart or Version 4.0+ of the Bluetooth).
- Run either iOS (version 8 or later) or Android OS (version 4.3 or later).
- Configured email-account.

Subjects may use their own compatible smartphone/tablet for use of the App. If, for any reason, the subject cannot or do not want to use their own mobile device they will be provided a preconfigured smartphone, as part of the study device kit, to be used during this study.

Use of subject's own mobile device smartphone

If the subject chooses to use his/her own mobile device the following steps should be followed at the Baseline visit before the subject leaves the site:

1. The study site personnel must ensure that the mobile device fulfils the requirements to use the App.
2. Download and open the App.
3. Open *Settings* and assign the study Subject ID as *Username* in the App.
4. Add the email address of the site personnel to receive reports from the subject.
5. Connect the App to the Navina Smart system unit assigned to the subject.
6. Site personnel to demonstrate how the App is used:
    a. Synchronization.
    b. Rating.
    c. NBD Score evaluation.
    d. Creating and sending reports.

Use of preconfigured smartphone in the study device kit

If a preconfigured smartphone supplied by WHC is to be used the following steps should be followed at the Baseline visit before the subject leaves the site:

1. Start the App, open *Settings* and assign the study Subject ID as *Username* in the App.
2. Ensure the email address of the site personnel to receive reports is entered correctly in the App.
3. Connect the App to the Navina Smart system assigned to the subject.
4. Site personnel to demonstrate how the App is used
    a. Synchronization.
    b. Rating.
    c. NBD Score evaluation.
    d. Creating and sending reports.

The smartphone must be returned to the study site at the subject's Termination Visit.

5.2 Procedures

The study device should only be used in this study and according to the Clinical Study Protocol (CSP).

For complete information regarding: preparation, activation, instillation, evacuation, disassembling and cleaning of the Navina Smart system, **see Appendix D; Instructions for use**. The Instructions for use (IFU) provides directions to help users and healthcare professionals to operate the Navina Smart system.

Navina Smart system helps patients with NBD symptoms to empty their bowels. When the catheter is inserted into the rectum, a small balloon fills with air to keep the catheter in place during intestinal irrigation. The balloon also functions as a seal inside the rectum to help retain the water as it is instilled into the bowel.

Process for initiation of the therapy will primarily be based on clinical judgment based on individual prerequisites. It is however recommended that the initiation is very careful to offer the best possible introduction for the patient. **Appendix C; Navina Treatment Schedule**, describes the recommended settings in tailoring and careful monitoring of the therapy to fit individual needs.

Subjects, who start to use Navina Smart, must be motivated and feel confident with the system. It is also essential that the subjects understand what TAI is and the risks involved. This is achieved by thorough information and training, together with proper follow-up and should include the following:

- *Anatomy and physiology of the bowel tract.*
- *The individual patient's bowel dysfunction as compared to normal bowel function.*
- *Information about what TAI is.*
- *TAI technique: hygiene, equipment, catheter insertion, emptying procedure, handling aids.*
- *The importance of regular bowel emptying with a deliberate program.*
- *Information about the risks involved and complications and where to turn to if they appear.*

- *Information on where to find more information concerning the training and training sessions, e.g. homepages, Navina-app, Navina training.*
- *Information about Navina Smart system and how it works.*

Designated staff at the participating centres will be trained by local Navina specialist in each country prior to study start. The study staff will subsequently train each study subject in the use of Navina Smart system. The training must be performed before any study devices are handed out to the subjects. All study subjects will be followed up, on a weekly basis by a telephone contact in which the therapist asks about performing TAI with the intention of identifying optimal performance and outcomes with irrigation. If there are specific problems reported by the patient, or there is inadequate efficacy of treatment, the therapist will advise tailoring of the TAI regime according to recommendations in Appendix C.

5.3 Treatment Compliance

For “inpatients” (patients who reside at a health care facility or equivalent); to ensure treatment compliance the local study personnel must follow the recommended manuals and the instructions in this protocol for the investigational product.

For “outpatients” (patients who live at home); at each scheduled site visit and phone contact subjects will be asked about compliance to study treatment. Once the subjects are enrolled in the study and are using the Navina Smart system for TAI they will, prior to the weekly phone follow-up, create reports (.pdf and.csv files) on irrigation and evaluation by synchronizing their Navina Smart unit with the Navina Smart app. These reports will be sent as email-attachments to the investigator/study site personnel and are to be used at the initial weekly telephone contacts to titrate/tailor an optimal TAI regimen.

5.4 Labelling

The study device is a commercially available product manufactured by WHC. In addition to the standard labelling (the commercially available), the boxes containing the study devices will be labelled as follows:

- “Exclusively for clinical investigation” in local language.
- Study code; “NAV-0001”.
- Name, address and contact information of the Principal Investigator.

5.5 Device Accountability

The study devices will not be distributed to the study site until all agreements between the principal investigator, the institution and WHC are finalized and applicable Ethical Committee (EC) and regulatory approvals (if applicable) have been obtained and communicated to WHC. All study devices must be kept in a secure place with controlled access, under appropriate storage conditions. A description of the appropriate storage and shipment condition are specified on the study product label.
Wellspect HealthCare will keep records to document the location of all study devices during the course of the study. Therefore, all product deliveries must be confirmed by the investigator or delegate. Furthermore, the investigator or delegate must keep records documenting the receipt, use, return and disposal of the study devices which shall include:

- Date of receipt at study site (logged in the Certificate of Delivery form).
- Identification of each study device (reference and LOT numbers, logged in the Certificate of Delivery form and in the eCRF).
- Expiry date (logged in the Certificate of Delivery form).
- Date(s) of use (logged in the eCRF).
- Subject identification (logged in the eCRF).
- Date of return of non-used, expired or malfunctioning study devices (logged in the Certificate of Return form).

All unused study products must be returned to WHC when treatment of the last subject has been completed. Any study product accidentally or deliberately destroyed must be accounted for and discrepancies between amounts dispensed and returned should be explained.

6 STUDY VARIABLES: DEFINITIONS AND ASSESSMENTS

6.1 Screening and Demographic Variables

The following screening and demographic data will be recorded by investigator or delegate into the eCRF:

- Date of birth.
- Sex.
- Race/Ethnicity.
- Relevant medical and surgical history.
- Medication at entry and during the study.
- Disease specific score, *i.e.* ASIA grade.

6.2 Primary and Secondary Outcome Variables

All outcome variables, both primary and secondary are listed in the table below:

Table 6 Outcome Variables

Objective	Outcome Variable	Method
To investigate change in NBD symptoms between baseline and 3 months use of Navina System (primary objective).	NBD score.	Analyses of absolute values and change between measurements according to instructions from the provider of the validated Neurogenic Bowel Dysfunction Score [15](NBD Score) at Baseline (Visit 1) and 3 months (Visit 2). **See section 6.4.1**

Table 6 Outcome Variables

Objective	Outcome Variable	Method
To investigate the change of QoL status in the selected patient population.	EQ-5D-3L.	Analyses of absolute values and change between measurements according to instructions from the provider (EuroQoL Group) [45]of EQ-5D-3L at baseline, 3 months and 12-months. **See section 6.4.2**
To investigate study product compliance.	PRO variables ("Do you still perform TAI with the Navina System?" Yes/No).	Descriptive analysis of data collected from patient questionnaires at 3 and 12 months. **See section 6.4.4**
To investigate NBD symptoms after 6, 9 and 12 months use of Navina™ System.	NBD Score.	Analyses of absolute values and change between measurements according to instructions from the provider of the validated NBD Score [15] at Baseline, 3, 6, 9 and 12 months. **See section 6.4.1**
To investigate patient perception and satisfaction of Bowel Management including TAI therapy and use of the Navina System.	PRO variables	Descriptive analysis of data collected from patient questionnaires at baseline, 3 months and 12 months. **See sections 6.4.3** and **6.4.4**
To investigate frequency of UTI.	PRO variables.	Descriptive analysis of data collected from patient questionnaires at baseline, 3 months and 12 months. **See sections 6.4.3** and **6.4.4**
To perform health economic analyses using QoL data and PRO variables.	EQ-5D-3L.	Analyses of absolute values and change between measurements according to instructions from the provider (EuroQoL Group) [45]of EQ-5D-3L at baseline, 3 months and 12-months. **See section 6.4.2**
	PRO variables.	Descriptive analysis of data collected from patient questionnaires at 3 and 12 months. **See section 6.4.4**

6.3 Safety Variables

The methods for collecting safety data are described below.

6.3.1 Adverse Events

6.3.1.1 Definitions

The definitions of Adverse Events (AEs), Device Deficiencies, Adverse Device Effects (ADEs), Serious Adverse Events (SAEs) and Serious Adverse Device Effects (SADEs) are provided below. It is of the utmost importance that all staff involved in the study are familiar with the content of this section. The principal investigator is responsible for ensuring this.

Adverse Event
An Adverse Event (AE) is defined as any untoward medical occurrence, unintended disease or injury, or untoward clinical signs (including abnormal laboratory findings) in subjects, users or other persons.
NB! This includes any and all events related to the study device, the comparator or the procedures involved.
NB! For users or other persons, this definition is restricted to events related to the study device.

Causality
The investigator is responsible for determining whether there is a causal relationship between an AE and the use of a medical device.
All AEs are categorized as unrelated, possibly related or related, as defined below:

- **Unrelated**: the AE is not reasonable in relation to the use of the medical device, or another cause can itself explain the occurrence of the event.
- **Possibly related**: the AE may be explained by the medical device and the onset is reasonable in relation to the use of the medical device, however there is insufficient information to determine the likelihood of this possibility.
- **Related**: the AE is most likely explained by the medical device and the onset is reasonable in relation to the use of the medical device.

An AE that is categorized as being **related** to the use of a medical device constitutes an ADE.

Device Deficiency
A device deficiency is defined as an inadequacy of a medical device with respect to its identity, quality, durability, reliability, safety or performance. This includes malfunctions, use errors, and inadequate labelling.

Adverse Device Effect
An Adverse Device Effect (ADE) is defined as an AE causally **related** to the use of a medical device. This definition also includes:

- Adverse events resulting from insufficient or inadequate instructions for use, deployment, implantation, installation, or operation, or any malfunction of the device.
- Any event resulting from use error or from intentional misuse of the study device.

Serious Adverse Event
A Serious Adverse Event (SAE) is an AE that fulfils one or more of the following criteria:

- Led to death.
- Led to a serious deterioration in the health of a subject, that either resulted in:
    - a life-threatening illness or injury, or
    - a permanent impairment of a body structure or a body function, or
    - in-patient or prolonged hospitalization (excluding hospital admissions and/or surgical operations that were planned before or during the study, where the illness/disease existed before the subject was enrolled and did not deteriorate in an unexpected way during the study), or
    - medical or surgical intervention to prevent life-threatening illness or injury or permanent impairment to a body structure or a body function.
- Led to foetal distress, foetal death or a congenital abnormality or birth defect.

Serious Adverse Device Effect
A Serious Adverse Device Effect (SADE) is defined as:

- An ADE that has resulted in any of the consequences characteristic of a serious adverse event, or
- A device deficiency that might have led to a serious adverse event if:
  - Suitable action had not been taken, or
  - An intervention had not been made, or
  - Circumstances had been less fortunate.

6.3.1.2 Adverse Event and Device Deficiency Reporting

Information about adverse events will be collected at each visit following the subject's signing of the informed consent throughout the treatment period.

Adverse Events
The investigator is responsible for recording the following in the CRF for **all** AEs:

- Adverse Event (i.e. the diagnosis or symptom(s))
- Start and stop date
- Whether the event is serious or not, and
- Causality
- Action taken with regard to study device
- AE Outcome

NB! If it is suspected that the medical device under study may have interfered with the effectiveness of a contraceptive medication/device, this should be reported as an ADE. Pregnancy itself, or elective abortions without complications, should not be reported as AEs.

Serious Adverse Events and Serious Adverse Device Effects

For **all serious** AEs, regardless of causality (i.e. both SAEs and SADEs), the investigator must:

- Complete an SAE report.
- Forward the AE and SAE reports, along with relevant supporting documents, to Wellspect HealthCare immediately but no later than the end of the next business day.
- If requested by Wellspect HealthCare, promptly follow up the initial SAE information with a detailed written report.
  NB! If the cause of death is reported as "unknown" the investigator must actively seek the

cause of death. If an autopsy has been performed, a copy of the results should be obtained (with translation of important parts into English).
- Inform the Ethics Committee of SAEs, as per local requirements (may include both those SAEs occurring at the study site as well as at other participating study sites, as well as cases when the opinion on seriousness/causality differs between him/her and Wellspect HealthCare.)

Device Deficiencies
The investigator is responsible for recording the following in the CRF for **all** device deficiencies:

- Device deficiency details
- *Start and stop* date, and
- Whether the device deficiency led to an adverse event

For **device deficiencies that fulfil the SADE definition** (see section 6.3.1.1 Serious Adverse Device Effect), the investigator must:

- Complete an extended Device Deficiency (SADE (Potential SAE)) report.
- Forward the Device Deficiency report, along with relevant supporting documents, to Wellspect HealthCare immediately but no later than the end of the next business day.
- If requested by Wellspect HealthCare, promptly follow up the initial information with a detailed written report.
- Inform the Ethics Committee of SADE (Potential SAE), as per local requirements (may include both those SADEs occurring at the study site as well as at other participating study sites, as well as cases when the opinion on seriousness/causality differs between him/her and Wellspect HealthCare.)

6.4 Patient-Reported Outcomes (PROs)

6.4.1 The Neurogenic Bowel Dysfunction score

To evaluate the effect of Navina System, a patient friendly version of the validated standardized NBD score [15] questionnaire will be used. The NBD score is a symptom-based score for neurogenic bowel dysfunction that takes into account both constipation and faecal incontinence, and weighs each symptom of NBD according to its impact on QoL in persons with spinal cord injury. The NBD score has a 10-item symptom severity scale to measure the parameters, faecal incontinence, constipation and obstructed defecation.
Each item is assigned a score based on odds ratios for associations between items and impact on QoL. Negative changes in NBD score correspond to an improvement in bowel-related symptoms, and the total NBD score can range from 0 (very minor bowel dysfunction) to 47 (severe bowel dysfunction).

The NBD score is self-administrated and will be completed by all subjects at baseline (visit 1), at 3 months follow-up (visit 2), in connection to the 6 and 9 months telephone follow-ups and at 12 months follow-up (Visit 3) or at end of the treatment period for those subjects with early withdrawal from the study. The subject will be provided with personal log-in details to *Viedoc™Me*, the patient interface of the eCRF, which will be used to complete the NBD score at baseline. The NBD score at 3, 6, 9 and 12 months follow-up will be completed using the Navina Smart app (see further instructions in 5.1.2). If, at any time during the study and for any reason,

the subject is unable to complete web-based questionnaires, the subject will be provided a paper questionnaire. Study site personnel will then enter the data into the study data base.

Applicable scores and results from the questionnaire will be analysed and presented according to the user instructions from the provider. The results from the NBD score at baseline and at the 3, 6, 9 and 12 months follow-up will be compared.

6.4.2 The EQ-5D-3L

EQ-5D is a standardized health-related quality of life questionnaire developed by the EuroQoL Group in order to provide a simple, generic measure of health for clinical and economic appraisal (EuroQoL Group, 1990)[45]. The five dimensions questionnaire (EQ-5D) is one of the most commonly used generic questionnaires to measure health-related quality of life (HRQOL). The EQ-5D is short, easy to use and flexible. It consists of a questionnaire and a visual analogue scale (VAS), i.e. EQ-VAS. The EQ-VAS is a self-rated health status using a VAS. The EQ-VAS records the subject's perceptions of their own current overall health and can be used to monitor changes with time. The questionnaire is a self-reported description of the subject's current health in 5 dimensions; *mobility, self-care, usual activities, pain/discomfort* and *anxiety/depression*. The subject is asked to grade their own current level of function in each dimension on a three-graded scale (i.e. no problems, some problems or severe problems). The combination of these creates a unique health state. Each health state can be ranked and transformed into a single score called the utility score, which can be used to estimate QALYs (Quality Adjusted Life Years) in health economic analyses. Due to its universality across diseases and patient populations, EQ-5D is one of the preferred methods to generate utilities for QALY estimation.

The EQ-5D-3L will be completed by all subjects at Baseline (visit 1), at the 3-months follow-up (visit 2) and at 12- months follow-up (Visit 3) or at end of the treatment period for those subjects with early withdrawal from the study. The subject will be provided with a paper questionnaire to be completed and the study site personnel will then enter the data into the eCRF.

Applicable scores and results from the questionnaire will be analysed and presented according to the user instructions from the provider (EuroQoL Group)[45]. The results from the EQ-5D-3L at Baseline, 3-months follow-up and 12- months follow-up will be compared as well as the change between the three assessments. The change in utility scores from baseline and the 3-months and 12 months follow-up, respectively, will be used for QALY calculations in health economic analyses.

6.4.3 The "Baseline Questionnaire"

The "Baseline Questionnaire" is a study-specific, non-validated instrument and is developed by WHC to provide measures of current bowel management, bowel dysfunction and frequency of UTI.

The "Baseline-Questionnaire" is self-administrated and will be completed by each subject only at Baseline (Visit 1). The subject will be provided with personal log-in details to *ViedocMe*, the patient interface of the eCRF, which will be used to complete the "Baseline Questionnaire". If, at any time during the study and for any reason, the subject is unable to complete web-based questionnaires, the subject will be provided with a paper questionnaire. Study site personnel will then enter the data into the study data base.

Applicable scores and results from the questionnaire will be analysed and presented using descriptive statistics.

6.4.4 The "Follow-up Questionnaire"

The "Follow-up Questionnaire" is a study-specific, non-validated instrument and is developed by WHC to provide measures that will reflect patient perception and satisfaction of bowel management, including TAI therapy and the use of Navina Smart system.

The Navina-specific questions relates to 5 dimensions; *Preparation, Activation, Instillation, Evacuation* and *Disassembling and Cleaning*. Additionally, the questionnaire contains questions to address product compliance and frequency of UTI.

The "Follow-up-Questionnaire" is self-administrated and will be given to all subjects at 3-months follow-up (Visit 2) and at 12- months follow-up (Visit 3) or at end of the treatment period for those subjects with early withdrawal from the study. The subject will be provided with personal log-in details to *ViedocMe*, the patient interface of the eCRF, which will be used to complete the "Follow-up Questionnaire". If, at any time during the study and for any reason, the subject is unable to complete web-based questionnaires, the subject will be provided a paper questionnaire. Study site personnel will then enter the data into the study data base.

Applicable scores and results from the questionnaire will be analysed and presented using descriptive statistics. Quantifiable data on healthcare utilization will be used in health economic analyses to assess changes in costs related to treatment with Navina Smart system.

7 STATISTICAL CONSIDERATIONS

7.1 Statistical Evaluation – General Aspects

Epidemiological methods will be used for the analysis of study data being a result of a non-interventional study design. Descriptive statistics, i.e. number of subjects, mean, median, standard deviation, minimum and maximum values for continuous data and frequencies and percentages for categorical data will be used as applicable. P-values below 5% will be considered statistically significant even though it is recognized that multiple hypotheses will be tested.

7.1.1 Demographics and Other Baseline Characteristics

Baseline data as well as other covariates will be presented by means of descriptive statistics and investigated by using epidemiological methods.

7.1.2 Covariates and Prognostic Variables

No considerations to potential covariates and/or prognostic factors are taken in the study design. Potential influence on any covariates and/or prognostic variables to the outcome of the study will be investigated in the statistical analysis using epidemiological methods.

7.1.3 Handling of Dropouts and Missing Data

Subjects dropping out will be compensated for in the sample size estimation. In case of higher drop-out than accounted for, it is recognized that recruitment may be extended to involve at least the minimum number of evaluable subjects since no risks for the subjects can be identified (i.e.

prescribed treatment are being observed). This is to ensure a representative sample for the purpose of the study/epidemiologic survey. Missing data will not be imputed.

7.1.4 Multi-Centre

This study is a multi-centre study. However, there is no a priori reason to suspect that there will be any qualitative differences between the study sites regarding any of the outcome variables in the study. Therefore, primary statistical analysis will not include centre in the model but subgroup analyses and health economic evaluations may use and present result by centre as applicable.

7.2 Description of Analysis Sets

The study will be analysed using a Per Protocol (PP) and an Intention To Treat (ITT) approach. Subjects fulfilling the inclusion criteria but none of the exclusion criteria and have been treated according to the protocol without any major deviations will be included in the PP analysis set. All correctly included patients will be included in the ITT analysis set. All treated subjects will be included in the safety analysis set. Treatment efficacy related conclusions will be based on the results from the PP analysis and confirmed by an analysis of the ITT analysis set (only for the primary objective) and demography and treatment safety related conclusions will be based on the results from the safety analysis set.

7.3 Method of Statistical Analysis in Relation to Objectives

Continuous data (data collected on a 5 grade scale will be regarded as continuous) will be analyzed using the non-parametric Wilcoxon Signed Rank Test. For data using a 2-graded scale the McNemar test will be used. Proportions are tested by the McNemar’s test if the number of non-equal answers between the assessment time-points is greater than 10 and by the binomial test if less than or equal to 10[46].

P-values below or equal to 5% will be denoted “statistically significant” even though it is recognized that multiple hypotheses are tested. The risk for multiplicity errors will be taken into account when conclusions are drawn.

7.4 Determination of Sample Size

This study aims to look at change in NBD score (range from 0 (very minor bowel dysfunction) to 47 (severe bowel dysfunction)) after introducing TAI with the Navina Smart system in a population with a common feature of having neurological bowel dysfunction. Each patient serves as his/her own control, by measuring change in NBD score before and after treatment with the Navina Smart system. The NBD score is a validated questionnaire and tool that correlates neurological bowel dysfunction to QoL [15]. In this study a patient friendly version of the NBD Score will be used.

To determine the sample size of the study the following formula is used, referred to in Lachin 1981, considering the fact that a pair wise comparison is made[47].

$$N = \frac{\left(Z_{\alpha} + Z_{\beta}\right)^2 \sigma^2}{\mu_1^2}$$

Where:
μ_1= minimal important difference or difference worth detecting

N = Total number of patients
Z_α = 1.96 (5% two-sided significance level)
Z_β = 1. 28 (1-β = 0.90), 90% power or 0.84 (1-β = 0.80), 80% power
σ = standard deviation of difference in NBD score between baseline (without Navina™) and 3 months use of Navina™.

The table below shows number of patients needed using different levels of minimal important difference and standard deviations, using 90% power and 5% two-sided significance level.

Total sample size for paired observations where σ is known and defined as standard deviation of difference between baseline and follow-up.

μ_1	σ= 2.0	σ= 2.1	σ= 2.2	σ= 2.5
**0.8**	66	73	80	103
**1.0**	43	47	51	66
**1.2**	30	33	36	46
**1.5**	19	21	23	30
**2.0**	11	12	13	17
**2.5**	7	8	9	11

The chosen minimal important difference for the study is related to the fact that also small difference could be clinically meaningful for the patients. In addition, depending on baseline NBD score level, smaller, but still valid, changes may be present when starting at a level closer to the inclusion cut of 10 than if the starting point is around 14. For this reason we believe a minimal important difference level valuable to detect in the study of ~0.8 would be representative.

Christensen et al describe in their sample published in 2008 a mean difference in NBD score of -4.5 with corresponding 95% confidence interval of -6.6 to -2.4 after introducing TAI for a 10 weeks' period[33]. This indicates that the expected standard deviation for the difference between baseline and follow up in NBD score would lie somewhere around 2.1-2.2. For detection of a minimal important difference of 0.8, with an expected standard deviation of 2.2, a total of 80 (90% power) subjects who has been using Navina Smart for 3 months are required. However, since higher variability than expected and high drop-out rates may risk the final numbers additional patients must be accounted for. For example, Gosselink et al. reported a discontinuation rate of 55% [48] when introducing TAI therapy in their sample reported in 2004 and Christensen et al. observed a discontinuation rate of 45% [49], why it is recommended to recruit at least 150 patients in the current study. This will allow 70 of 150 (47%) to drop-out from the study with maintained 90% power to detect a difference of 0.8 between a baseline NBD score (without Navina Smart) and after using Navina Smart for 3 months.

7.5 Statistical Analyses During the Course of the Study

Estimates on minimal important difference and standard deviation may be checked as part of an adaptive statistical design and conditional sample size estimation by interim analysis of a selection of patients, e.g. approximately 60 patients. If the variability is found to be higher and/or the minimal important difference is found to be lower the study is at risk of being inconclusive. In such case additional subjects or premature discontinuation may be considered.
Once the target number of evaluable subjects is secured, i.e. 90% power of the primary variable is verified, partial clean file and analysis of the primary variable and baseline demography will be performed. It is acknowledged that analyses of primary variables and baseline demography will be performed before analyses of secondary variables and analyses of the complete data set. All

analyses will follow the Data Management Plan and be performed on locked data, declared for partial clean file or clean file for the complete data set.

8 DATA MANAGEMENT

Data Management activities will be performed by subjects, site personnel and WHC or their representatives. See Appendix E for an overview of the data flow in the study.

8.1 Electronic Data Capture

Data Entry into a central database will be made using Electronic Data Capture (EDC).

Data Entry will mainly be performed by the subjects, but will be performed by study site personnel in cases where EDC is not possible for the subject, *i.e.* where questionnaires are completed using pen and paper (e.g. all EQ-5D-3L forms), for Screening and Demographic variables as well as for AEs and Concomitant Medication.

Data will be entered into eCRFs using Viedoc, a web based EDC system used by WHC. Trained and authorized study site personnel will be responsible for entering data into Viedoc. Data entered into Viedoc will be immediately saved to a central database, hosted by a 3rd party, Pharma Consulting Group Solutions AB.

Study site personnel will enrol subjects using Viedoc. Upon enrolment in the study each subject will be automatically allocated a specific Subject ID (Enrolment number). This is the only identifier used on any study data and the key (the Subject Enrolment and Identification Log) connecting a specific subject to his/her Subject ID will be kept by the Investigator in the Investigator's Study File which is stored securely at the study site.

Subjects will use a site-specific tablet computer to self-report ePRO data using ViedocMe. ViedocMe is the patient interface application of the eCRF and subject specific log-in details will be provided to the subject by site personnel. Upon completion of an ePRO the data will be automatically entered into Viedoc and thus stored in the central database.

The EQ-5D forms will be completed in paper by the subjects.

Subjects unable to complete ePRO will use pen and paper instead. If pen and paper are used, trained authorized study site personnel will enter the data into Viedoc.

8.1.1 Navina Smart App Data

The Navina Smart app is running locally on the mobile device and all data associated with the App is stored on a local database. No transferring of data to an external database for central storage is performed, except for anonymous data that is stored for the purpose of data analytics in order to improve the product and the therapy. The App may run without Internet connectivity, even though the creation and sending of reports require internet access.

The Navina Smart app communicates with the Navina Smart unit via Bluetooth Smart protocol, and a checksum is used to validate the correctness of the data transferred. The App has no limits of number of irrigation data to be stored, other than the capacity of the mobile device storage.

The Navina Smart app and its data will stay on the mobile device regardless of the user upgrading the App or the operating system. At deletion of the Navina Smart app, data is deleted, except from anonymous data transferred to Wellspect HealthCare.

Reports sent by email from an App user to site personnel will consist of .pdf and .csv files. The email with the attached reports will automatically contain the Subject ID. The .csv files contain irrigation data and NBD Score data and will be uploaded by site personnel into the subject's eCRF.

8.2 Validation

Data validation will be performed by WHC or its representative.

Data queries will be raised for inconsistent, impossible or missing data. Comments made in Viedoc or ViedocMe in any other language than English will be queried for translation, unless already translated by study site personnel. The study site personnel are required to resolve any such queries. All entries to the study database will be available in an audit trail.

When data has been entered, reviewed, edited and, if applicable, source data has been verified, the data will be locked to prevent further editing and, if not already done, the principal investigator will be prompted to sign the eCRF electronically. Readable copies of the eCRF data, stored on a CD-ROM or a DVD will be archived in the ISF at the study site after Clean File.

The study data will be validated as defined in the Data Management Plan. When all data have been validated, clean file will be declared and the database will be locked.
Study data will be securely stored at WHC, with restricted access.
Data will be retained at WHC 25 years after study closure.

9 STUDY MANAGEMENT

9.1 Monitoring

Before the first subject is enrolled into the study, a WHC representative will visit the study site to:

- Determine the adequacy of the facilities.
- Discuss with the investigator(s) (and other personnel involved with the study) their responsibilities with regard to protocol adherence, and the responsibilities of WHC or its representatives. This will be documented in a Clinical Study Agreement between WHC and the investigator.

During the study, a monitor from WHC or company representing WHC will have regular contacts with the study site, including visits to:

- Provide information and support to the investigator(s).

- Confirm that facilities remain acceptable.
- Confirm that the study site team is adhering to the protocol, that data are being accurately recorded in the CRFs and that study devices are accounted for.
- Perform source data verification (a comparison of the data in the CRFs with the subject's medical records at the hospital or practice, and other records relevant to the study). This will require access to the medical records for each subject (e.g., clinic charts).

A WHC representative will be available between visits if the investigator(s) or other staff at the study site needs information and advice.

9.2 Audits and Inspections

Authorized WHC representatives, a regulatory authority and/or an Ethics Committee may visit the study site to perform audits or inspections, including source data verification. The purpose of a WHC audit or inspection is to systematically and independently examine all study-related activities and documents to determine whether these activities were conducted, and data were recorded, analyzed, and accurately reported according to the clinical study protocol, ISO 14155 Good Clinical Practices and any applicable regulatory requirements. The investigator should contact WHC immediately if contacted by a regulatory authority about an inspection at his or her study site.

9.3 Training of participating centres - Staff

To ensure sufficient knowledge in use and handling of the Navina System, study site personnel who will conduct the training of subjects at each site will firstly be trained by local WHC representative.

The principal investigator must maintain a record of all individuals involved in the study (medical, nursing and other staff), i.e. a Delegation of Responsibilities form. He or she shall ensure that appropriate training relevant to the study is given to all of these staff, and that any new information of relevance to the performance of this study is forwarded to the staff involved.

Before data of the first subject is entered into the study, the study staff will also be trained to use the chosen EDC system by WHC personnel or delegates.

9.4 Changes to the Protocol

Study procedures will not be changed without the mutual agreement of the principal investigator and WHC.

If it is necessary for the study protocol to be amended, the amendment and/or a new version of the study protocol (Amended Protocol) must be notified to or approved by each Ethics Committee, and if applicable, also the local regulatory authority, before implementation. Local requirements must be followed.

If a protocol amendment requires a change to a particular centre's Informed Consent Form, then WHC and the centre's Ethics Committee must be notified. Approval of the revised Informed Consent Form by WHC and by the Ethics Committee is required before the revised form is used.

WHC will distribute administrative changes, amendments and new versions of the protocol to each principal investigator.

9.5 Deviations from the Protocol

The investigator is not allowed to deviate from the Clinical Study Protocol except in emergency situations, with purpose to protect a subject's rights, safety and wellbeing.

In such cases, the investigator may proceed without prior approval of the sponsor and the EC. Furthermore, such deviations shall be documented and reported as soon as possible to the sponsor and the EC.

Should the investigator break any obligations under the Clinical Study Protocol or Clinical Study Agreement (including a failure without just cause to meet a timeline) and fail to remedy such a breach where it is capable of cure, WHC retains the right to disqualify the study site from further study participation.

9.6 Study Agreements

The principal investigator at each study site should comply with all the terms, conditions, and obligations of the Clinical Study Agreement for this study. In the event of any inconsistency between this Clinical Study Protocol and the Clinical Study Agreement, the terms of Clinical Study Protocol shall prevail with respect to the conduct of the study and the treatment of subjects and in all other respects, not relating to study conduct or treatment of subjects, the terms of the Clinical Study Agreement shall prevail.

Agreements between HC and the principal investigator should be in place before any study-related procedures can take place, or subjects are enrolled.

9.7 Before Enrolment

Before a subject's enrolment in the study and any study-related procedures are undertaken the following should be fulfilled:

- Signed Clinical Study Protocol and other agreements between WHC and the principal investigator/study site.
- Approval of the study by the Ethics Committee.
- Approval of the study, if applicable, by the regulatory authority.

9.8 Early Termination of the Study

The end of the study is defined as ‘the last visit of the last subject undergoing the study’.
The study may be terminated at individual study sites if the study procedures are not being performed according to regulations (Declaration of Helsinki, ISO 14155 and applicable regulatory requirements) or if recruitment is slow. WHC may also terminate the entire study prematurely if concerns for safety arise within this study or in any other study with the study device.

For more information regarding subject follow-up at discontinuation of study participation, see Section 4.5.2.

9.9 Publication Policy

WHC is committed to communicate product, research and development information in an accurate and objective fashion according to external standards regarding the manner and content of scientific, technical and medical publications. WHC is committed to provide patients and

healthcare professionals with meaningful information about the trials WHC sponsor and about WHCs products to enable them to make the best treatment decisions.

Wellspect HealthCare's commitment is met by providing details of the study and its results on the publicly accessible website http://www.clinicaltrials.gov/. WHC uses a unique trial identifier (i.e., study code) for website postings and in all subsequent publications.

Furthermore, WHC seeks to ensure that publications in biomedical journals follow the guidelines established by the International Committee of Medical Journal Editors (ICMJE) and published in its *Uniform Requirements of Manuscripts Submitted to Biomedical Journals*. Importantly, these guidelines require recognition in the manuscript that the research was conducted in accordance with the *Ethical Principles for Medical Research Involving Human Subjects*, otherwise known as the *Declaration of Helsinki*.

Publication, authorship and use of study results are regulated in the Clinical Study Agreement for this study.

10 ETHICS

10.1 Ethics Review

The final study protocol, including the final version of the Informed Consent Form, must be approved or given a favourable opinion in writing by an Ethics Committee (EC) as appropriate. The investigator must forward the written Ethics Committee approval to Wellspect HealthCare before he or she can enrol any subject into the study.

The Principal Investigator is responsible for informing the Ethics Committee of any amendment to the protocol, in accordance with local requirements. In addition, the Ethics Committee must approve all advertising used to recruit subjects for the study.
All EC approvals must be forwarded to WHC, before implementation.
The Principal Investigator is also responsible for providing the Ethics Committee with reports of any SAEs/SADEs from any other study conducted with the study device. WHC will provide this information to the Principal Investigator.

Progress reports and notifications of SAEs/SADEs will be provided to the Ethics Committee according to local regulations and guidelines.

10.2 Ethical Conduct of the Study

The study will be performed in accordance with ethical principles that have their origin in the Declaration of Helsinki and are consistent with ISO 14155 and applicable local regulatory requirements.

10.3 Informed Consent

The principal investigator at each study site will:

- Obtain informed consent from each subject before any study-related procedures are initiated. This includes any procedure or assessment required to confirm a subject's eligibility for the study, which is outside of the subject's routine medical care.

- Ensure each subject is given full and adequate oral and written information about the nature, purpose, possible risk and benefit of the study (using non-technical, understandable language).
- Ensure that the subject is informed that an Ethics Committee has approved all aspects of the study, including the written information.
- Make clear that neither the principal investigator nor the study site personnel are allowed to force or improperly influence a subject to participate, or to continue to participate, in the study.
- Ensure that the subject understands that he/she may withdraw from the study at any time without this affecting his/her future care.
- Ensure that the subject understands that he/she allows access to personal data by regulatory authorities and WHC representatives by signing the Informed Consent Form.
- Provide the subject with the Informed Consent Form and with adequate opportunity to ask questions and adequate time for consideration before deciding upon participation in the study.
- Ensure that the Informed Consent Form is signed and dated by the subject and by the principal investigator/delegate responsible for the consenting process.
- Provide the subject with a copy to keep and file the original in the Investigator's Study File.
- Inform already included subjects of any changes to the Clinical Study Protocol that affect study participation and obtain each subject's dated signature on the new version of the Informed Consent Form, if updated.

10.4 Subject Data Protection

The Informed Consent Form will incorporate wording that complies with relevant data protection and privacy legislation. Pursuant to this wording, subjects will authorize the collection, use and disclosure of their study data by the investigator and by those persons who need that information for the purposes of the study.

The Informed Consent Form will explain that study data will be stored in a computer database, maintaining confidentiality in accordance with national data legislation. All data computer processed by WHC will be identified only by the study code NAV-0001 and the unique Subject ID.

The Informed Consent Form will also explain that for data verification purposes, authorized representatives of WHC, a regulatory authority, an Ethics Committee may require direct access to parts of the hospital or practice records relevant to the study, including subjects' medical history.

10.5 Insurance

Subjects participating in this clinical study are insured by WHC, provided that the Clinical Study Protocol and other written procedures are adhered to. For further details, see the Clinical Study Agreement.

11 STUDY CONTACTS

Table 7 Study Contacts

Role in the study	Name	Address & telephone number
Clinical Study Team Leader	Susanne Lindholm	Wellspect HealthCare<br>P.O. Box 14<br>SE-431 21 Mölndal<br>Sweden<br>Phone: +46 31 376 4503<br>Fax: +46 31 376 4212
Clinical Study Team Member	Annika Asting	Address as above.<br>+46 31 376 4736
Clinical Study Team Member	Lina Berggren	Address as above.<br>Phone: +46 31 376 4319
Clinical Study Team Member	David Antonsson	Address as above.<br>Phone: +46 31 3763151
Director Medical Affairs and Clinical Education	Anna-Karin Sundin	Address as above.<br>Phone: +46 31 376 3292

In the case of a medical emergency you should contact the Clinical Study Team Leader. If the Clinical Study Team Leader is not available, contact the Director of Medical Affairs and Clinical Education.
The principal investigator is responsible for ensuring that procedures and expertise are available to handle medical emergencies during the study. A medical emergency usually constitutes an SAE and should be reported as such, see Section 6.3.1.2.

12 REFERENCES

1. Harari D, Norton C, Lockwood L, Swift C. Treatment of constipation and faecal incontinence in stroke patients: randomized controlled trial. *Stroke; a journal of cerebral circulation.* Nov 2004;35(11):2549-2555.
2. Faaborg PM, Christensen P, Kvitsau B, Buntzen S, Laurberg S, Krogh K. Long-term outcome and safety of transanal colonic irrigation for neurogenic bowel dysfunction. *Spinal cord.* Jul 2009;47(7):545-549.
3. Emmanuel A. Review of the efficacy and safety of transanal irrigation for neurogenic bowel dysfunction. *Spinal cord.* Sep 2010;48(9):664-673.
4. Naicker AS, Roohi SA, Naicker MS, Zaleha O. Bowel dysfunction in spinal cord injury. *The Medical journal of Malaysia.* Jun 2008;63(2):104-108.
5. Roach MJ, Frost FS, Creasey G. Social and personal consequences of acquired bowel dysfunction for persons with spinal cord injury. *The journal of spinal cord medicine.* Winter 2000;23(4):263-269.
6. Coggrave M, Norton C, Wilson-Barnett J. Management of neurogenic bowel dysfunction in the community after spinal cord injury: a postal survey in the United Kingdom. *Spinal cord.* Apr 2009;47(4):323-330; quiz 331-323.
7. Menter R, Weitzenkamp D, Cooper D, Bingley J, Charlifue S, Whiteneck G. Bowel management outcomes in individuals with long-term spinal cord injuries. *Spinal cord.* Sep 1997;35(9):608-612.
8. Valles M, Vidal J, Clave P, Mearin F. Bowel dysfunction in patients with motor complete spinal cord injury: clinical, neurological, and pathophysiological associations. *The American journal of gastroenterology.* Oct 2006;101(10):2290-2299.
9. Stiens SA, Bergman SB, Goetz LL. Neurogenic bowel dysfunction after spinal cord injury: clinical evaluation and rehabilitative management. *Archives of physical medicine and rehabilitation.* Mar 1997;78(3 Suppl):S86-102.
10. Singal AK, Rosman AS, Bauman WA, Korsten MA. Recent concepts in the management of bowel problems after spinal cord injury. *Advances in medical sciences.* 2006;51:15-22.
11. Glickman S, Kamm MA. Bowel dysfunction in spinal-cord-injury patients. *Lancet.* Jun 15 1996;347(9016):1651-1653.
12. Anderson KD. Targeting recovery: priorities of the spinal cord-injured population. *Journal of neurotrauma.* Oct 2004;21(10):1371-1383.
13. Liu CW, Huang CC, Yang YH, Chen SC, Weng MC, Huang MH. Relationship between neurogenic bowel dysfunction and health-related quality of life in persons with spinal cord injury. *Journal of rehabilitation medicine : official journal of the UEMS European Board of Physical and Rehabilitation Medicine.* Jan 2009;41(1):35-40.
14. Krogh K, Nielsen J, Djurhuus JC, Mosdal C, Sabroe S, Laurberg S. Colorectal function in patients with spinal cord lesions. *Diseases of the colon and rectum.* Oct 1997;40(10):1233-1239.
15. Krogh K, Christensen P, Sabroe S, Laurberg S. Neurogenic bowel dysfunction score. *Spinal cord.* Oct 2006;44(10):625-631.
16. Krogh K, Christensen P, Laurberg S. Colorectal symptoms in patients with neurological diseases. *Acta neurologica Scandinavica.* Jun 2001;103(6):335-343.
17. Paris G, Gourcerol G, Leroi AM. Management of neurogenic bowel dysfunction. *European journal of physical and rehabilitation medicine.* Dec 2011;47(4):661-676.
18. Emmanuel AV, Krogh K, Bazzocchi G, et al. Consensus review of best practice of transanal irrigation in adults. *Spinal cord.* Oct 2013;51(10):732-738.

19. McClurg D, Hagen S, Hawkins S, Lowe-Strong A. Abdominal massage for the alleviation of constipation symptoms in people with multiple sclerosis: a randomized controlled feasibility study. *Mult Scler.* Feb 2011;17(2):223-233.
20. Shafik A, El-Sibai O, Shafik IA. Physiologic basis of digital-rectal stimulation for bowel evacuation in patients with spinal cord injury: identification of an anorectal excitatory reflex. *The journal of spinal cord medicine.* Winter 2000;23(4):270-275.
21. Korsten MA, Singal AK, Monga A, et al. Anorectal stimulation causes increased colonic motor activity in subjects with spinal cord injury. *The journal of spinal cord medicine.* 2007;30(1):31-35.
22. Bove A, Pucciani F, Bellini M, et al. Consensus statement AIGO/SICCR: diagnosis and treatment of chronic constipation and obstructed defecation (part I: diagnosis). *World journal of gastroenterology : WJG.* Apr 14 2012;18(14):1555-1564.
23. Christensen P, Krogh K. Transanal irrigation for disordered defecation: a systematic review. *Scandinavian journal of gastroenterology.* May 2010;45(5):517-527.
24. Rubesin SE, Levine MS, Laufer I, Herlinger H. Double-contrast barium enema examination technique. *Radiology.* Jun 2000;215(3):642-650.
25. Shandling B, Gilmour RF. The enema continence catheter in spina bifida: successful bowel management. *Journal of pediatric surgery.* Mar 1987;22(3):271-273.
26. Mowatt G, Glazener C, Jarrett M. Sacral nerve stimulation for faecal incontinence and constipation in adults. *Cochrane Database Syst Rev.* 2007(3):CD004464.
27. Malone PS, Ransley PG, Kiely EM. Preliminary report: the antegrade continence enema. *Lancet.* Nov 17 1990;336(8725):1217-1218.
28. Gerharz EW, Vik V, Webb G, Leaver R, Shah PJ, Woodhouse CR. The value of the MACE (Malone antegrade colonic enema) procedure in adult patients. *Journal of the American College of Surgeons.* Dec 1997;185(6):544-547.
29. Siddiqui AA, Fishman SJ, Bauer SB, Nurko S. Long-term follow-up of patients after antegrade continence enema procedure. *Journal of pediatric gastroenterology and nutrition.* May 2011;52(5):574-580.
30. Krassioukov A, Eng JJ, Claxton G, Sakakibara BM, Shum S. Neurogenic bowel management after spinal cord injury: a systematic review of the evidence. *Spinal cord.* Oct 2010;48(10):718-733.
31. Christensen P, Kvitzau B, Krogh K, Buntzen S, Laurberg S. Neurogenic colorectal dysfunction - use of new antegrade and retrograde colonic wash-out methods. *Spinal cord.* Apr 2000;38(4):255-261.
32. Christensen P, Bazzocchi G, Coggrave M, et al. A randomized, controlled trial of transanal irrigation versus conservative bowel management in spinal cord-injured patients. *Gastroenterology.* Sep 2006;131(3):738-747.
33. Christensen P, Bazzocchi G, Coggrave M, et al. Outcome of transanal irrigation for bowel dysfunction in patients with spinal cord injury. *The journal of spinal cord medicine.* 2008;31(5):560-567.
34. Koch SM, Uludag O, El Naggar K, van Gemert WG, Baeten CG. Colonic irrigation for defecation disorders after dynamic graciloplasty. *International journal of colorectal disease.* Feb 2008;23(2):195-200.
35. Koch SM, Melenhorst J, van Gemert WG, Baeten CG. Prospective study of colonic irrigation for the treatment of defaecation disorders. *The British journal of surgery.* Oct 2008;95(10):1273-1279.
36. Christensen P, Krogh K, Buntzen S, Payandeh F, Laurberg S. Long-term outcome and safety of transanal irrigation for constipation and faecal incontinence. *Diseases of the colon and rectum.* Feb 2009;52(2):286-292.

37. Christensen P, Krogh K, Perrouin-Verbe B, et al. Global audit on bowel perforations related to transanal irrigation. *Techniques in coloproctology.* Feb 2016;20(2):109-115.
38. Loffeld RJ, Engel A, Dekkers PE. Incidence and causes of colonoscopic perforations: a single-center case series. *Endoscopy.* Mar 2011;43(3):240-242.
39. Panteris V, Haringsma J, Kuipers EJ. Colonoscopy perforation rate, mechanisms and outcome: from diagnostic to therapeutic colonoscopy. *Endoscopy.* Nov 2009;41(11):941-951.
40. Atkin WS, Cook CF, Cuzick J, Edwards R, Northover JM, Wardle J. Single flexible sigmoidoscopy screening to prevent colorectal cancer: baseline findings of a UK multicentre randomised trial. *Lancet.* Apr 13 2002;359(9314):1291-1300.
41. Meeting notes, ISCOS 2013, Coloplast Symposium on Peristeen TAI. 2013.
42. MAUDE on Peristeen. 2015. http://www.accessdata.fda.gov/scripts/cdrh/cfdocs/cfmaude/search.cfm. Accessed Dec 31 2015.
43. Valles M, Benito J, Portell E, Vidal J. Cerebral hemorrhage due to autonomic dysreflexia in a spinal cord injury patient. *Spinal cord.* Dec 2005;43(12):738-740.
44. Khastgir J, Drake MJ, Abrams P. Recognition and effective management of autonomic dysreflexia in spinal cord injuries. *Expert opinion on pharmacotherapy.* May 2007;8(7):945-956.
45. Group. TE. EuroQol-a new facility for the measurement of health related quality of life. *Health Policy.* 1990;16:199-208.
46. Agresti A. *An Introduction to Categorical Data Analysis.* first ed: John Wiley & Sons; 1996.
47. Lachin JM. Introduction to sample size determination and power analysis for clinical trials. *Controlled clinical trials.* Jun 1981;2(2):93-113.
48. Gosselink MP, Darby M, Zimmerman DD, et al. Long-term follow-up of retrograde colonic irrigation for defaecation disturbances. *Colorectal disease : the official journal of the Association of Coloproctology of Great Britain and Ireland.* Jan 2005;7(1):65-69.
49. Christensen P, Olsen N, Krogh K, Bacher T, Laurberg S. Scintigraphic assessment of retrograde colonic washout in faecal incontinence and constipation. *Diseases of the colon and rectum.* Jan 2003;46(1):68-76.
