# Supplementary material for: An open prospective study on the efficacy of Navina Smart, an electronic system for transanal irrigation, in neurogenic bowel dysfunction
Source: PLoS One. 2021 Jan 29;16(1):e0245453. doi: 10.1371/journal.pone.0245453 (PMC7845961; doi:10.1371/journal.pone.0245453)
Supplement: S1 Appendix — Introduction to transanal irrigation (TAI) is a highly individual process. The Navina™ Smart system settings will benefit the tailoring of the therapy to fit individual needs. This treatment schedule indicates what steps to take when making modifications of the study subject’s irrigation therapy. (PDF) [file pone.0245453.s003.pdf]

Wellspect®
A REAL DIFFERENCE

NAVINA SMART TREATMENT SCHEDULE

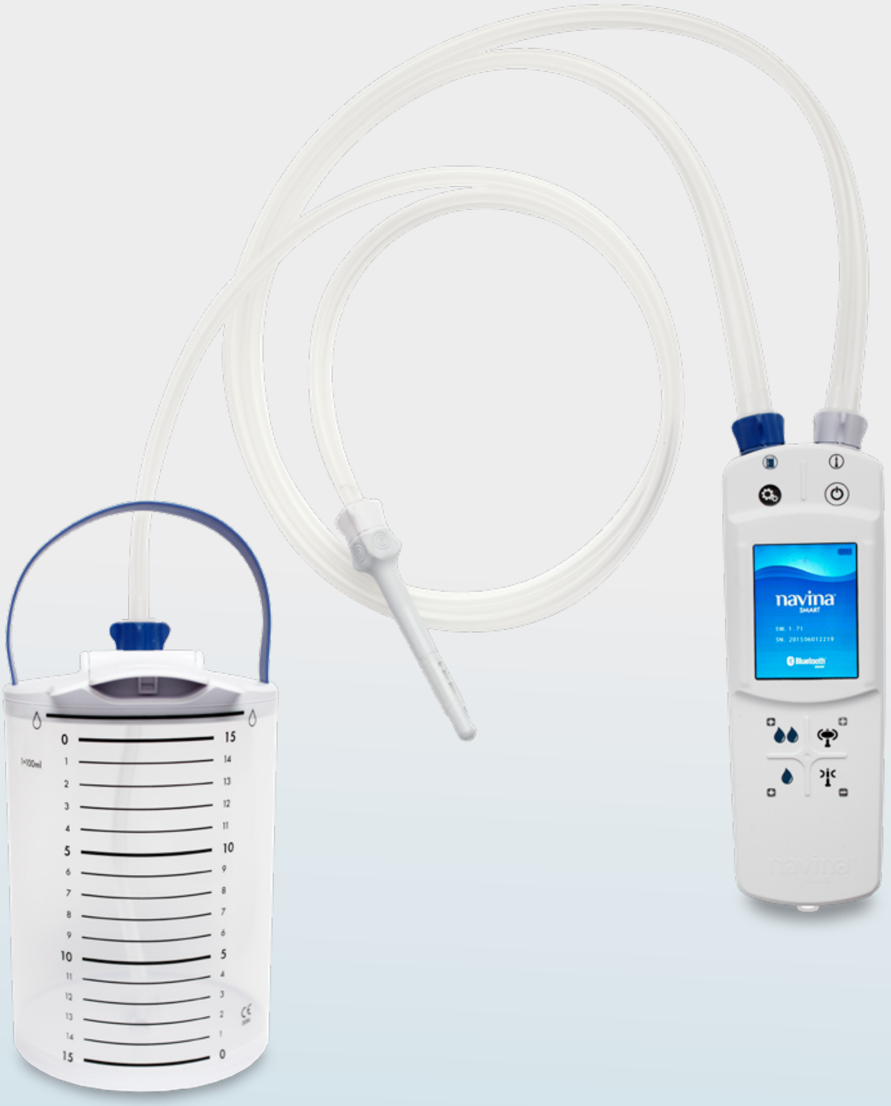

Navina™ Smart

Version: 1.0

Introduction to transanal irrigation (TAI) is a highly individual process. The Navina™ Smart system settings will benefit the tailoring of the therapy to fit individual needs. This treatment schedule indicates what steps to take when making modifications of the study subject's irrigation therapy.

INITIATION OF TAI THERAPY

The process for initiation of the therapy will primarily be based on clinical judgment based on individual prerequisites. It is however recommended that the initiation should offer the best possible introduction for the subject. The following settings should be used to support careful initiation of TAI therapy:

1. **Frequency** – Every day as starting point. It is recommended to irrigate after awakening or after a meal when the bowel contractions are the best.

2. **Balloon size** – Use level 2 (corresponding to 40,5 ± 5 mm) in order to minimize trauma and avoid reflex emptying with bigger balloon size.

3. **Volume** – Initially use 700 ml of water, water temperature 36-38°C.

4. **Flow rate** – Setting 2, corresponding to 200 ml/min.

MODIFICATIONS TO THERAPY

The weekly follow-up phone calls will be initiated by the therapist with the intention of identifying optimal performance and outcome of the irrigation.

If specific problems/symptoms are reported by the subject, or there is inadequate efficacy of treatment, the therapist will advise tailoring of the TAI regime according to recommendations below.

If the subject is satisfied with existing settings, continue as is. If problems/symptoms described below occur, follow recommendations for each problem/symptom. If the subject reports more than one problem/symptom, then define and deal with the most troublesome for the subject.

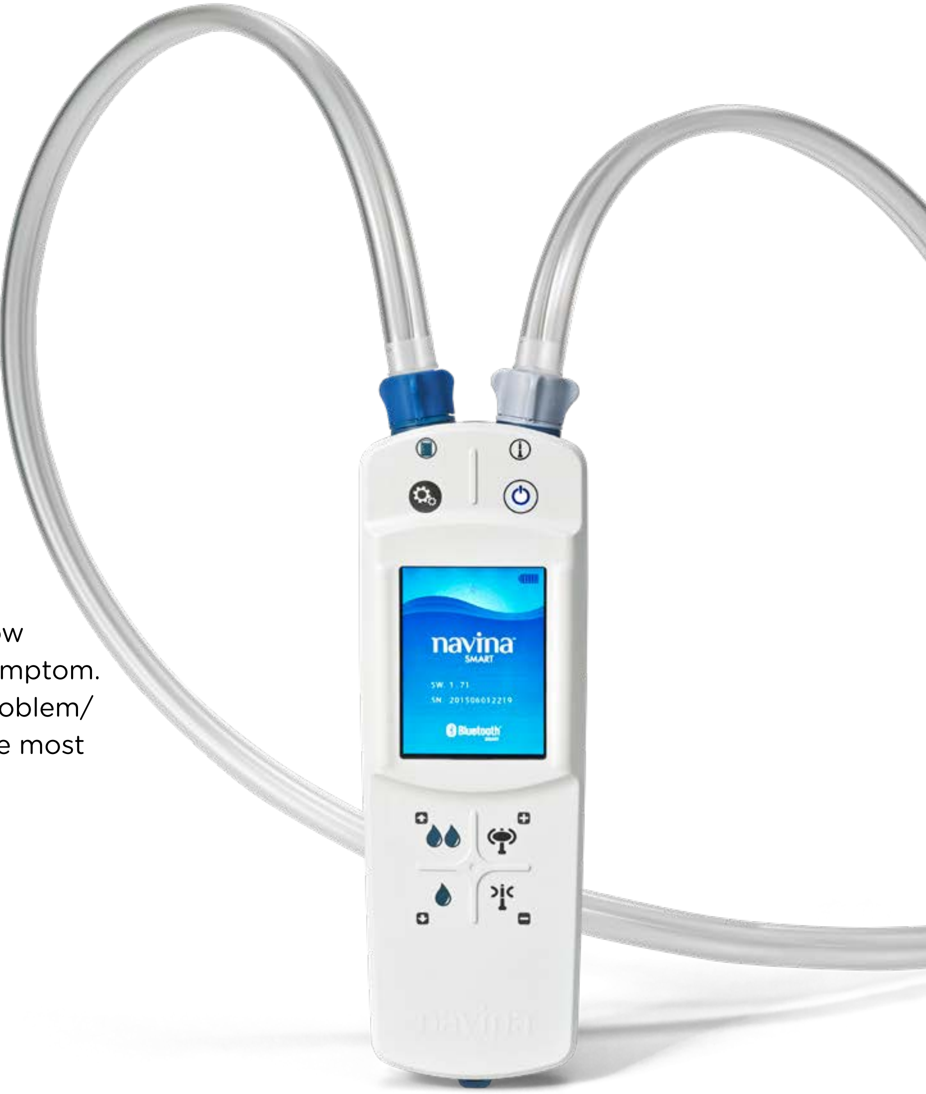

TREATMENT SCHEDULE TAI WITH NAVINA SMART

1. **Initiation**
   TAI every day
   Balloon size, setting 2
   Flowrate 200 ml/min
   700 ml of water

2. **Weekly phone follow-up**

3. **Subject satisfaction**
   - **YES** → 3A. Continue with existing settings
   - **NO** → 3B. Modify settings
     See following pages...

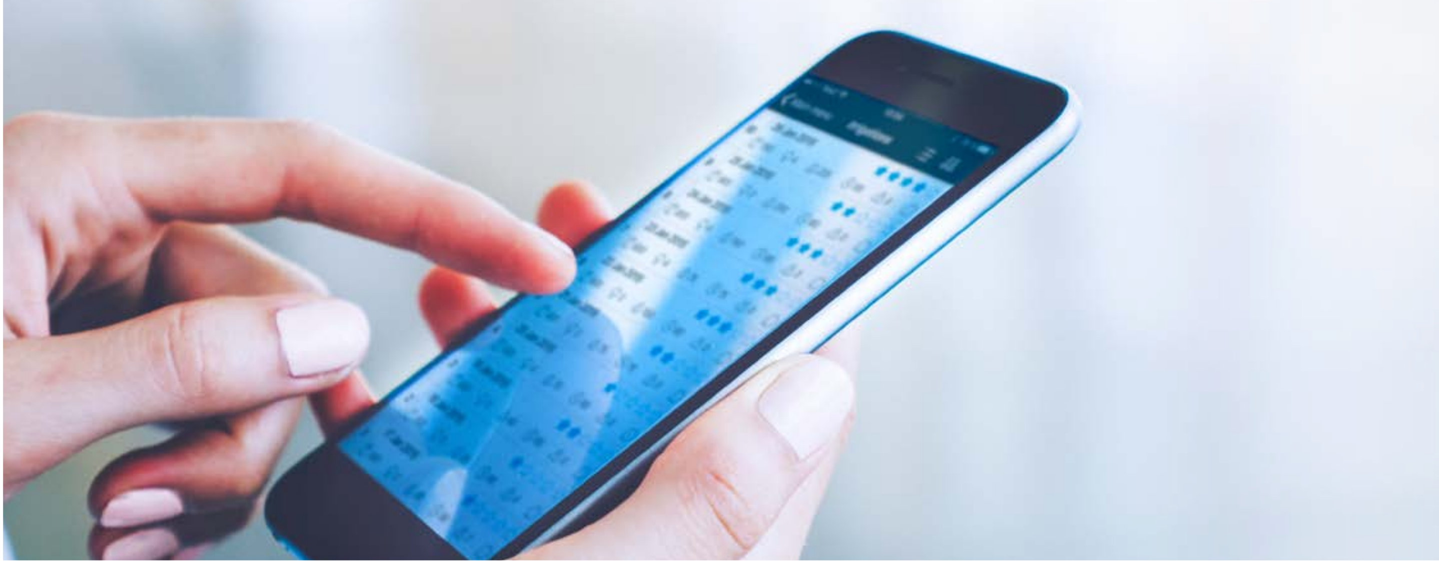

Version: 1.0

3B. MODIFY SETTINGS

(most concerning symptom)

Rectal bleeding and pain

Bleeding

- A small amount of blood seen on the catheter is not a concern.
- More copious or regular bleeding requires the subject to come back promptly for clinical assessment.
- Frank haemorrhage with or without pain suggests a probable perforation which should be treated as a medical emergency.

Pain

1. If cramps, discomfort or pain occur while instilling the irrigation fluid, ensure that water is warm enough – at body temperature, 36–38°C.
2. If pain continues, pause instillation for a few seconds and reduce flow rate of irrigation to 50 % (i.e. if 200 ml/min is used step down to 100 ml/min) once the discomfort has subsided.
3. In case of severe or persistent pain, stop irrigating – possible bowel perforation (especially if associated with bleeding) – assess as a potential medical emergency.

Autonomic dysreflexia and autonomic symptoms

(sweating, palpitations, dizziness)

1. Slow down flow rate of irrigation by 50 % (e.g. if 200 ml/min is used step down to 100 ml/min).
2. If dysreflexia persists, reduce volume being irrigated with 50 % (e.g. if 500 ml is used step down to 250 ml).

Leakage of water around the catheter

1a. Ensure catheter is properly located.

1b. Ensure water temperature is 36-38°C.

2. Increase balloon inflation by choosing the next level in the settings, e.g. if setting 1 (31,5 ± 5 mm) is used increase to setting 2 (40,5 ± 5 mm).
3. Slow down rate of irrigation by decreasing the flow rate by 50 %, i.e. if 200 ml/min is used step down to 100 ml/min.

3B. MODIFY SETTINGS

(most concerning symptom)

Reflex expulsion of the catheter

1a. Ensure water temperature 36–38°C.

1b. Digital check to ensure rectum empty of stool.

2. Reduce rate of balloon inflation by releasing the button for balloon inflation.
3. Decrease balloon size, e.g. if setting 2 (40,5 ± 5 mm) is used decrease to setting 1 (31,5 ± 5 mm).
4. Suggest clinical reassessment to check for and treat constipation.

Difficulty inserting catheter or instilling irrigation fluid

1. Digital rectal check and removal of stool if present.
2. Increase frequency of TAI to ensure evacuation is adequate. E.g. if TAI is used every second day, increase to daily use.
3. Increase volume of water with 100 ml to ensure evacuation is adequate.

Faecal incontinence and leakage of irrigant

Faecal incontinence between uses of TAI

- Increase volume of water by small increments (100 ml) until satisfactory evacuation achieved with no faecal incontinence.
- Split the irrigation into 2 consecutive episodes, 10-15 minutes between episodes, using half the water each time.
- Increase frequency of TAI.
- Decrease frequency of TAI.
- Consider laxative use.

3B. MODIFY SETTINGS

(most concerning symptom)

Irrigant is not expelled

1a. Use adjunctive measures to treat constipation.

1b. Confirm subject is adequately hydrated.

2. Repeat irrigation with 50 % of volume (all other parameters unchanged), e.g. if 500 ml is used, change to 250 ml.

No stool is evacuated after TAI

1a. Ensure irrigation has been performed as instructed.

1b. Use adjunctive measures to treat constipation.

1c. Ensure the subject is adequately hydrated.

2. No stool may be present if a good result was obtained at last irrigation; if this happens regularly reduce frequency of irrigation.
3. If no stool for several days, suspect constipation/impaction, assess and re-assess subject and treat accordingly.
4. Increase volume of water with 100 ml. Continue to increase in 100 ml increments.
5. Increase flow rate by choosing the next step in the settings, e.g. if setting 2 (200 ml/min) is used increase to setting 3 (300 ml/min).

Leakage of water between irrigations

- Ensure subject allows sufficient time on toilet following TAI.
- Encourage use of adjunctive measures to encourage emptying.
- Reduce amount of water instilled.
- Increase amount of water instilled.
- Split the irrigation into 2 consecutive episodes, 10–15 minutes between episodes, using half the water each time.
- Increase frequency of TAI.
- Decrease frequency of TAI.
- An anal plug can be tried if problem persists.

At Wellspect we value the people behind our success as a leading provider of life-changing products for bowel and bladder management such as the well-known brands LoFric® and Navina™. From the thousands of users and healthcare professionals worldwide who inspire our innovative solutions, we know that working together is the best way to advance continence care, giving our users more time for life. Building on over 30 years of life-improving performance, we passionately strive to make a difference every day to everyone who needs our products and services.

Wellspect. A Real Difference.

wellspect.com

Wellspect HealthCare, Aminogatan 1, P.O. Box 14, SE-431 21 Mölndal, Sweden. Phone: +46 31 376 40 00.

Version: 1.0
