# Supplementary material for: An open prospective study on the efficacy of Navina Smart, an electronic system for transanal irrigation, in neurogenic bowel dysfunction
Source: PLoS One. 2021 Jan 29;16(1):e0245453. doi: 10.1371/journal.pone.0245453 (PMC7845961; doi:10.1371/journal.pone.0245453)
Supplement: S1 Questionnaire — (PDF) [file pone.0245453.s004.pdf]

Study code: NAV-0001

Subject ID: |__|__|-|__|__|

Follow-up Questionnaire

(3 Months)

Assessment date: |__|__|__|__|__|__|__|__|

YYYY MM DD

3-MONTH FOLLOW-UP QUESTIONNAIRE

Dear study participant,

Thank you for taking the time to evaluate the Navina™ Smart system.

At this visit you will evaluate the Navina Smart system after 3 months use.

The questionnaire is self-administrated, however, if you are unsure about some of the questions, please ask your study physician or study nurse.

Paper version: Mark the options given under each question which you believe best describes your situation. When you are asked to answer in more detail, please write as clearly as possible. There are no "right" or "wrong" answers.

Thank you for your participation.

COMPLIANCE

1. Are you still using Navina Smart as part of your bowel management?

☐ No
☐ Yes (proceed to question **2**)

a) If **No**, specify the reason(s) why you do not still use Navina Smart. Tick all that apply.

☐ Too difficult inserting catheter or instilling irrigant
☐ No stool evacuated after transanal irrigation
☐ Leakage of irrigation fluid around the catheter during irrigation
☐ Leakage after/between transanal irrigation (TAI) procedure(s)
☐ General discomfort
☐ Handling difficulties
☐ Too time-consuming
☐ Ineffective treatment
☐ Trauma to anus and/or rectum due to balloon burst
☐ Bleeding
☐ Pain
☐ Autonomic dysreflexia
☐ Other, specify: ________________________________

Even though you have stopped using Navina Smart (if answered "No") we would like you to complete the rest of this questionnaire to get your opinion on the use and handling of the system.

BOWEL FUNCTION

2. Which method(s), other than TAI, do you usually use as a part of your current bowel management? Tick all that apply.

☐ Diet and fluids
☐ Stool softeners
☐ Spontaneous/voluntary bowel emptying
☐ Digital rectal stimulation
☐ Digital removal of faeces
☐ Micro-enema laxatives
☐ Rectal suppository laxatives
☐ Leaning forward during bowel emptying
☐ Massage of abdomen
☐ None
☐ Other, specify: ________________________________

Wellspect HEALTHCARE

3. Specify the type of problem(s) you experience with your current bowel management. Tick all that apply.

☐ Constipation
☐ Diarrhoea
☐ Bowel accidents/faecal incontinence
☐ Abnormal bloating or cramping pain
☐ None
☐ Other, specify: ____

4. How satisfied are you in general with your current bowel management?

☐ Completely satisfied
☐ Satisfied
☐ Neutral
☐ Not satisfied
☐ Not satisfied at all

5. How much time daily do you spend on your current bowel management (approximately)?

__________________Hours __________________Minutes

6. How often do you visit the toilet in order to empty, or try to empty, your bowels (approximately)?

__________________________ times/week

7. At how many of these visits do you actually manage to empty your bowels (approximately)?

__________________________ times/week

8. How much time do you spend, on average, **sitting on the toilet** at each of these visits (approximately)?

__________________Hours __________________Minutes

9. How satisfied are you in general with TAI as a therapy?

☐ Completely satisfied
☐ Satisfied
☐ Neutral
☐ Not satisfied
☐ Not satisfied at all

INSTRUCTIONS AND TRAINING

10. Do you feel that you initially received adequate instructions and training in how to perform TAI with Navina Smart?

☐ No
☐ Yes

11. Specify the amount of training in performing TAI with Navina Smart that you initially received (approximately):

__________________Hours __________________Minutes

12. Specify the number of supervised TAI sessions you initially performed with your healthcare professional (doctor/nurse): _____________

FAECAL INCONTINENCE AID(S)

13. Do you use incontinence aid(s) (for example shields, undergarments, sanitary towels, sheets, diapers etc.)?

☐ No (proceed to question **14**)
☐ Yes

a) If **Yes**, specify average number of pads/absorbent products or underwear changes required per week: ______________

GENERAL HANDLING OF THE NAVINA SYSTEM

14. Do you handle the Navina Smart system yourself (majority of the time)?

☐ No
☐ Yes (proceed to question **15**)

a) If **No**, specify the type of assistance required during the irrigation procedure. Tick all that apply. I need assistance with:

☐ The entire TAI procedure
☐ Setting up the system
☐ Filling the water container
☐ Catheter insertion
☐ Catheter withdrawal
☐ Operation of Navina Smart unit
☐ Disassembling the system
☐ Cleaning the system
☐ Using the Navina Smart app
☐ Other, specify: ____

b) If **No**, estimate required time of assistance in total per week.

Note that this refers only to assistance related to the irrigation procedure, NOT assistance required in daily life.

___________________Hours ___________________Minutes

15. I find using the Navina Smart system to be:

- ☐ Very practical
- ☐ Practical
- ☐ Neutral
- ☐ Not so practical
- ☐ Not practical at all

HANDLING – BEFORE INSERTION

16. I find handling of the packaging to be:

- ☐ Very easy
- ☐ Easy
- ☐ Neutral
- ☐ Difficult
- ☐ Very difficult
- ☐ I do not know

17. I find setting up the Navina Smart system (connecting tubes, catheter, water container etc.) to be:

- ☐ Very easy
- ☐ Easy
- ☐ Neutral
- ☐ Difficult
- ☐ Very difficult
- ☐ I do not know

18. I find handling of the water container to be:

- ☐ Very easy
- ☐ Easy
- ☐ Neutral
- ☐ Difficult
- ☐ Very difficult
- ☐ I do not know

19. I find activation (wetting) of the Navina rectal catheter to be:

☐ Very easy
☐ Easy
☐ Neutral
☐ Difficult
☐ Very difficult
☐ I do not know

HANDLING – AT INSERTION

20. I find the Navina Smart unit display/symbols to be:

☐ Very easy
☐ Easy
☐ Neutral
☐ Difficult
☐ Very difficult
☐ I do not know

21. I find insertion of the Navina rectal catheter to be:

☐ Very easy
☐ Easy
☐ Neutral
☐ Difficult
☐ Very difficult
☐ I do not know

HANDLING – DURING IRRIGATION

22. Do you add anything to the irrigation fluid (water)?

☐ No (proceed to question **23**)
☐ Yes, sometimes
☐ Yes, always

a) If **Yes**, specify what you add to your irrigation fluid (water). Tick all that apply.

☐ Phosphate
☐ Polyethylene glycol
☐ Table salt
☐ Soap
☐ Laxatives
☐ Other, specify: ______________________________

23. Have you experienced any involuntary rectal catheter expulsions since you started using the Navina Smart system?

☐ No (proceed to question **24**)
☐ Yes

a) If **Yes**, specify how many involuntary rectal catheter expulsions you have experienced:

____

24. Do you experience leakage of irrigation fluid around the catheter during irrigation (when the fluid is instilled)?

☐ No
☐ Yes, sometimes
☐ Yes, always
☐ I do not know

HANDLING – AFTER IRRIGATION

25. I find handling/deflation of the balloon to be:

☐ Very easy
☐ Easy
☐ Neutral
☐ Difficult
☐ Very difficult
☐ I do not know

26. I find handling of the rectal catheter at withdrawal to be:

☐ Very easy
☐ Easy
☐ Neutral
☐ Difficult
☐ Very difficult
☐ I do not know

27. I find disassembling the Navina Smart system after irrigation to be:

☐ Very easy
☐ Easy
☐ Neutral
☐ Difficult
☐ Very difficult
☐ I do not know

28. I find cleaning of the Navina Smart system to be:

☐ Very easy
☐ Easy
☐ Neutral
☐ Difficult
☐ Very difficult
☐ I do not know

TAI PROCEDURE – <u>FLUID AND STOOL EVACUATION</u>

29. After catheter withdrawal, does **the irrigation fluid** evacuate immediately from the bowel?

☐ No
☐ Yes (proceed to question **30**)

a) If **No**, specify average time for fluid evacuation:

___________________Hours ___________________Minutes

30. After catheter withdrawal, does **the stool** evacuate immediately from the bowel?

☐ No
☐ Yes (proceed to question **31**)

a) If **No**, specify average time for stool evacuation:

___________________Hours ___________________Minutes

31. How often do you need to repeat the irrigation procedure during the same visit to the bathroom in order to ensure emptying of the bowel?

☐ Never
☐ Less than once a week
☐ Once a week
☐ More than once a week
☐ Always

URINARY TRACT INFECTION (UTI)

32. Have you experienced any symptomatic UTI **during the previous 3 months**?

A symptomatic UTI is when urinary symptoms (e.g. discomfort or pain over the kidney or bladder or on passing urine, onset of urinary incontinence or fever) have been experienced and a diagnosis of UTI has been made by a health professional. This may or may not have been treated with antibiotics.

☐ No (proceed to question **33**)
☐ Yes

a) If **Yes**, specify number of UTIs during the previous 3 months: ______________________

b) Did you receive prescription of antibiotics?

☐ No (proceed to question **33**)
☐ Yes

c) If **Yes**, specify number of antibiotic courses due to UTI that you received during the previous 3 months: _________________

PERCEPTION – NAVINA SMART APP

33. I find synchronising the Navina Smart unit with the Navina Smart app to be:

☐ Very easy
☐ Easy
☐ Neutral
☐ Difficult
☐ Very difficult
☐ I do not know

34. I find creating/sending reports in the Navina Smart app to my healthcare professional to be:

☐ Very easy
☐ Easy
☐ Neutral
☐ Difficult
☐ Very difficult
☐ I do not know

35. I find using the Navina Smart app to be:

☐ Very easy
☐ Easy
☐ Neutral
☐ Difficult
☐ Very difficult
☐ I do not know

36. I think the Navina Smart app makes me feel in control as I get continuous feedback on the progress.

☐ Strongly agree
☐ Somewhat agree
☐ Neither agree nor disagree
☐ Somewhat disagree
☐ Strongly disagree
☐ I do not know

PERCEPTION – NAVINA SMART SYSTEM

37. Please specify how satisfied you are with the Navina Smart system.

☐ Completely satisfied
☐ Satisfied
☐ Neutral
☐ Not Satisfied
☐ Not Satisfied at all

38. I think the electronic operation of the Navina Smart system makes it easy for me to perform TAI.

☐ Strongly agree
☐ Somewhat agree
☐ Neither agree nor disagree
☐ Somewhat disagree
☐ Strongly disagree
☐ I do not know

39. I think the electronic display makes me feel in control as I get continuous feedback on the progress.

☐ Strongly agree
☐ Somewhat agree
☐ Neither agree nor disagree
☐ Somewhat disagree
☐ Strongly disagree
☐ I do not know

40. I think using Navina Smart makes me feel secure as the settings will keep my treatment at the prescribed level.

☐ Strongly agree
☐ Somewhat agree
☐ Neither agree nor disagree
☐ Somewhat disagree
☐ Strongly disagree
☐ I do not know

Thank you.

You have now completed all questions in this questionnaire.
